# Crash severity analysis and risk factors identification based on an alternate data source: a case study of developing country

**DOI:** 10.1038/s41598-022-25361-5

**Published:** 2022-12-08

**Authors:** Hanif Bhuiyan, Jinat Ara, Khan Md. Hasib, Md Imran Hossain Sourav, Faria Benta Karim, Cecilia Sik-Lanyi, Guido Governatori, Andry Rakotonirainy, Shamsunnahar Yasmin

**Affiliations:** 1 Queensland University of Technology, CARRS-Q, Queensland, Australia; 2 Data61, CSIRO, Brisbane, Australia; 3 Department of Electrical Engineering and Information System, University of Pannonia, Veszprem, Hungary; 4 Department of CSE, Ahsanullah University of Science and Technology, Dhaka, Bangladesh; 5 Memorial University of Newfoundland, St. John’s, Canada; 6 Department of EEE, University of Asia Pacific, Dhaka, Bangladesh; 7 Centre for Computational Law, Singapore Management University, Singapore

**Keywords:** Civil engineering, Computer science

## Abstract

Road traffic injuries are one of the primary reasons for death, especially in developing countries like Bangladesh. Safety in land transport is one of the major concerns for road safety authorities and other policymakers. For this reason, contributory factors identification associated with crashes is necessary for reducing road crashes and ensuring transportation safety. This paper presents an analytical approach to identifying significant contributing factors of Bangladesh road crashes by evaluating the road crash data, considering three different severity levels (non-fetal, severe, and extremely severe). Generally, official crash databases are compiled from police-reported crash records. Though the official datasets are focusing on compiling a wide array of attributes, an assorted number of unreported issues can be observed that demands an alternative source of crash data. Therefore, this proposed approach considers compiling crash data from newspapers in Bangladesh which could be complimentary to the official crash database. To conduct the analysis, first, we filtered the useful features from compiled crash data using three popular feature selection techniques: chi-square, Two-way ANOVA, and Regression analysis. Then, we employed three machine learning classifiers: Decision Tree, Random Forest, and Naïve Bayes over the extracted features. A confusion matrix was considered to evaluate the proposed model, including classification accuracy, sensitivity, and specificity. The predictive machine learning model, namely, Random Forest using Label Encoder with chi-square and Two-way ANOVA feature selection process, seems the best option for crash severity prediction that provides high prediction accuracy. The resulting model highlights nine out of fourteen independent features as responsible factors. Significant features associated with crash severities include driver characteristics (gender, license type, seat belts), vehicle characteristics (vehicle type), road characteristics (road surface type, road classification), environmental conditions (day of crash occurred, time of crash), and injury localization. This outcome may contribute to improving traffic safety of Bangladesh.

## Introduction

Road crashes are responsible for more than 1.3 million deaths, whereas additional 50 million people are severely injured or permanently disabled across the world every year^1,2^. Through significant interactive efforts, developed countries have already reduced the number of crashes and associated casualties. However, an opposite trend is observed in most of the developing countries, and the situation is worsening over time. Bangladesh, a developing country with annual growth of 8.3% Gross domestic product (GDP), is not an exception.

In Bangladesh, more than eight people are reported to die in road crashes every day. Based on official crash records of the year 2016, 3412 people were reported to be fatally injured, and additional 8572 people were severely injured from road crashes. In the following year, the number of casualties increased to 4284 fatalities and 9112 serious injuries^3^. The Bangladesh Passengers Welfare Association (BPWA) announced that 6686 people lost their lives, and 8600 people were injured in 4891 road crashes in Bangladesh amid COVID-19-related lockdown in 2020^4^. However, in Bangladesh, the actual rate of road crash-related fatalities is significantly underreported^3^. The increasing trend in crash records clearly shows the urgent need to identify and implement countermeasures to prevent these unfortunate events. A way forward towards devising effective countermeasures is to identify the responsible factors for these unfortunate events based on a data-driven and evidence-based approach. As such, the primary focus of this study is to identify the relevant features of crash severity outcomes by using crash data from Bangladesh.

Analysis of crash data has mostly been depended on the official crash database compiled by different regions, and these databases are generally compiled from police-reported crash records. To date, these traditional crash databases provide with most detailed crash information, including crash characteristics, vehicle characteristics, roadway data, and other situational attributes. However, these databases are likely to be associated with underreporting, specifically for lower injury severity categories. In developing countries, both severe and non-severe crashes are likely to go underreported. Underreporting of crash data is not only a challenge in low- and middle-income countries but also an ongoing data collection challenge among several developed countries^5–7^. Given such a challenge, researchers and practitioners often identify an alternate source of crash data compilation.

In Bangladesh, a significant number of crashes go underreported in the official crash database. While accessibility to crash data is often imposed for privacy concerns, it might be worth exploring other data sources for developing countries. To be sure, any data collection is prohibitively expensive, and hence, the identification of alternate data sources can not only complement the official crash database but also can reduce the cost of data recording (to a certain extent). Moreover, an alternate data source can serve as a complementary source for addressing the significant underreporting issue of the crash database for developing countries like Bangladesh. In recent times, multiple studies considered online media data (such as news websites, social media platforms, several websites, etc.) and government official crash reports for crash analysis of developed countries. According to Pervaz et al. crash data reported by police, print media, and national and international organizations are significant to understand the potential magnitudes and current trends^8^. However, the scenario in developing or under-developing countries like Bangladesh is different. For instance, social media platforms are not enriched for getting crash data in developing or under-developing countries. Government websites mostly share fatal crashes report, which is also a scenario of underreported data for medium or lower injury data. Nowadays, several Bangladeshi newspapers have emphasized reporting crashes considering every category, including information about the crash area, time, pedestrian type, number of deaths, injury, and other relevant damage that make this platform a potential source of crash data to ensure the analysis result unbiased. Besides, Siddik et al. mentioned that collected crash data from the newspaper (like ‘Prothom Alo’) is a valuable source for analysis and predicting the death ratio in developing countries like Bangladesh^9^.

Therefore, it is worth mentioning that newspapers might be valuable resources to collect crash reports for traffic safety analysis in Bangladesh. As such, in this study, we have identified crash data compiled from Newspapers as an alternate source of crash data compilation. Specifically, we develop and propose a data scrapping algorithm in developing a compiled crash database from different crash characteristics compiled in these articles.

In this study, we have compiled crash records reported in several newspapers of Bangladesh for the year 2019. We opted for the most famous and oldest newspapers among several newspapers: Daily Prothom Alo, Daily Jugantor, and Bdnews24 (details could be found in “Study design”). The detailed information on different crashes reported in this newspaper is collected and compiled from the e-archive of these newspapers. This research is limited to the identified factors from these news articles.

Moreover, by using the compiled database, we also aim to study the risk factors responsible for road crashes in Bangladesh. Specifically, this paper presents an analytical approach to identify significant contributing factors of Bangladesh road crashes by evaluating the road crash data, considering three different severity levels (non-fatal, severe, and extremely severe injury outcomes). First, we employ three popular feature selection techniques (chi-square, Two-way ANOVA, and Regression analysis) for filtering out the useful features. Then, we employ three machine learning classifiers: Decision Tree, Random Forest, and Naïve Bayes (both multinomial and gaussian naïve bayes) over the extracted features to analysis the crash severity level. A confusion matrix is considered to evaluate the proposed model, including classification accuracy, sensitivity, and specificity. It is worth mentioning that the newspaper-based crash data is highly likely to be biased towards more severe crashes, and these data records will also be subjected to underreporting. Therefore, the findings solely based on these data records should be interpreted cautiously. It is beyond the scope of this study to examine the validity of such newspaper-archived crash databases. The major focus of this study is to identify an alternative approach toward compiling crash data and develop the data scrapping algorithm while also analyzing those data to identify the critical factors relevant to these crashes. The validation of compiled data against the official data source is beyond the proposed approach. The developed data scrapping framework is generic and can be adopted for further studies. Validating or augmenting these data with the official crash data might be an avenue for future research. Our major focus is on analyzing the data reported in newspapers, which could be a potential source of an alternate crash database to complement the official crash database with limited resources in developing countries like Bangladesh.

The rest of the paper is organized as follows. “Background study” represents the related past studies in the crash severity analysis area. In “Study design”, we present our data through data collection and data processing. “Crash Severity analysis and responsible factors prediction” explains the proposed crash severity analysis and risk factors identification model with system architecture. The model evaluation with several statistical analyses presents in “Evaluation Result”. “Result and discussion” shows the analysis result with elaborative discussion. In “List of crash factors” and “Implications”, we describe our identified crash risk factors and add some implications, respectively. Finally, we conclude the work through a conclusion in section “Conclusion and future worK”.

## Background study

Predicting crash severity and identifying the responsible factors are two significant issues of traffic safety research. Therefore, various approaches have been developed and implemented for crash severity prediction and identification of influential factors. A summary of the most recent related research on crash severity analysis and prediction is provided in Table 1. The information presented in Table 1 includes the research method, severity level, features, performance metrics, and prediction results. A detailed review of all the relevant studies is beyond the scope of this study. In summarizing the previous literature results, we have mostly focused on safety studies employing machine learning-based approaches while also considering a few studies from traditional statistical approaches for comparison purposes.

**Table 1:** Summary of previous crash severity analysis and prediction studies.

Previous studies	Methodological strategy	Crash severity representation	Feature selection techniques	Considered features	Significant responsible features	Performance prediction	Performance metrics
Zhang et al. (2021)	Negative binomial regression model Random effects negative binomial regression model	High/low-risk road segment	No	**Temporal characteristics—**cross winds, poor alignment, severe road damage, and transition to transportation facilities such as tunnels	Road segment/damage	NBR and RENBR model Low-risk 58% High-risk 71.8%	Coef., St.Err., t-value, p value
Rezapour et al. (2021)	Binary logistic regression Classification tree	Serious and fatal crashes	Yes	**Driver characteristics—**gender age, residency, speed limit compliance, driver conditions **Vehicle characteristics—**vehicle maneuver, traffic, and the number of vehicles **Environmental characteristics—**weather conditions, road conditions, and lighting conditions, day of a crash, weekend or weekday, and time of crash **Roadway characteristics—**crash location include vertical and horizontal characteristics of the segment and the posted speed limit of the segment	BLR**—**alcohol involvement, non-speed compliance road surface conditions, CT**—**speed limit	BLR: Actual**—**30% misclassification rate CV**—**31% misclassification rate CT: Actual**—**30% misclassification rate CV**—**32% misclassification rate	p value, confusion matrix (TP, FP, TN, FN), Precision, Recall, Specificity and Accuracy Feature: RFE algorithm
Delen et al. (2017)	Artificial neural networks Support vector machine Decision trees Logistic regression	Low or high Level of injury	No	**Driver characteristics—**drugs and/or alcohol levels, seatbelt, gender/age **Roadway characteristics—**road type/situation, direction, strike versus struck, number of cars and/or other objects involved, road surface condition **Environmental characteristics—**weather conditions, visibility and/or light conditions, time of the day **Vehicle characteristics—**the age of the vehicle, weight of the vehicle, body type of the vehicle	**S**eat belt, manner of collision, ejection, and drug	ANN**—**85.77% Accuracy SVM**—**90.41% Accuracy DT**—**86.61% Accuracy LR**—**76.97% Accuracy	Confusion matrices, accuracy, sensitivity, specificity AUC score
Huting et al. (2016)	Random Forest Logistic Regression	Accident Non-accident	No	**Trip characteristic—**trip type, trip distance, route hours, trip season **Environmental characteristic—**average snow depth, temperature, working shift, Traffic, Gender, Operator working day **Route characteristic—**route frequency, route type, previous accident	RF**—**age (40–65), Trip distance (10 mi), Operator working day (previous day) LR-older, inexperienced and female operators	RF—68.5% accuracy LR—72% accuracy	–
Mafi et al. (2018)	C4.5 Instance-based Random Forest	No-injury Minor injury, Severe injury	No	**Vehicle characteristics—**vehicle type, physical defects **Road characteristics—**roadway/traffic (Speed limit, Work zone, Area type, AADT, Road width, Road surface) **Environment characteristics—**hour, day of week, month, weather, light **Driver characteristics—**seat belt, air bag, age/gender (older female (O-F), older male (O-M), younger female (Y-F), and younger male (Y-M))	Area type, Road width, seat belt, younger female (Y-F), and younger male (Y-M)	C4.5**—**75.7% IB**—**82.8% RF**—**87.15%	–
Uddin and Huynh (2020)	Mixed Logit models	Major injury (fatality and disabling injury) Minor injury (evident injury and possible injury), No injury	No	**Environment characteristic—**weather (normal, rain and snow), speed, lane, time, weekend **Driver characteristics—**male/female, seat belt **Crash characteristics—**Rural, urban, curve, rear-end, sideswipe, object, MVIT, daylight, dark-lighted, dark-unlighted, rear-end **Vehicle characteristics—**single-unit truck, truck trailer, truck semi-trailer	Rain and snow, male, dark-lighted, time, rear-end	Confidence level-99%	Degree of freedom (df), p values, t-statistic, p2 values, standard deviation (sd)
Yahaya et al. (2020)	Firth logistic regression model	Fatal injury Non-fatal injury	No	**Driver characteristic—**age: (18–35), (36–50), (> 50), gender (male, female) **Type of construction—**base, asphalt, remove asphalt, milling, concrete **Environment characteristic-**weather (dry, fog, rain), speed limit: 90 km/h, 40 km/h, time period:(0–6), (6–9), (9–15), (15–18), (18–24), Weekday (Sunday–Saturday), Month (January–December) **Road character—**straight and level, curve, U-turn, straight and grade, lane width, road class (rural, urban) **Crash characteristic—**rear end, fixed object, sideswipe, angle, pedestrian	lane width, road character (U-turn)	FLR**—**0% mislabels	AUC-ROC statistic
Pillajo-Quijia et al. (2020)	Random Forest Classification and regression tree Support vector machine	DKSI: driver Killed and seriously injured DSI: Driver Slightly Injured	Yes	**Driver characteristics—**trip purpose, action of driver, driver license, psychophysical conditions, infractions for speeding, driver's infractions, Planned trip, driver seatbelt use, location of serious injury, driver age, driver gender **Vehicle characteristics—**vehicle age, condition of vehicle, group of light trucks and vans, occupants involved, gross vehicle weight **Road infrastructure—**road function, lane width, shoulder type, accident location **Environmental condition—**sight distance, lighting condition, weather, crash time, month/season	Driver characteristics (license, psychophysical conditions (alcohol, drugs, or sleep deprivation), seatbelt, driver age and gender)	RF**—**77% accuracy CART—78% accuracy SVM—79% accuracy RF + CART—70% accuracy	Accuracy, sensitivity, specificity, ROC area Feature: Gini index value
Lin et al. (2020)	Random Forest XGBoost	Not injured, non-incapacitating injury Possible injury, suspected serious injury, Killed	No	**Road characteristic—**road class, roadway type, speed limit, number of lanes, traffic control type **Driver characteristic—**person age, person restraint used **Temporal characteristic—**left shoulder use, construction zone, right shoulder type, first harmful event **Environment condition—**light condition, school zone, weather condition, manner of collision	Road class (Highways, City Street, Interstate etc.), speed limit, and the first harmful event (Pedestrian, Animal, Pedal cyclist etc.)	XGBoost-MAE (0.7140) Random Forest**—**MAE (0.7271)	Mean absolute error (MAE)
Ghandour et al. (2020)	Sequential minimal optimization Random Forest Artificial neural network Logistic Regression Naïve Bayes	Fatal Non-fatal	Yes	**Crash characteristic—**crash date and time (i.e., month, weekday, hour), location **Vehicle type—**motorcycle, truck, bike, pedestrian **Road type—**motorway, primary, secondary, tertiary **Temporal characteristic-**injury severity level (no apparent injury, minor injury, serious injury), and the number of fatalities	Crash type, injury severity, spatial cluster-ID, and crash time (h)	F1 score (SMO**—**0.493, RF**—**0.453, ANN**—**0.385, LR**—**0.455, NB**—**0.313) AUC-PR (SMO**—**0.276, RF**—**0.376, ANN**—**0.291, LR**—**0.361, NB**—**0.337) Kappa (SMO**—**0.4678, RF**—**0.4258, ANN**—**0.3462, LR**—**0.4309, NB**—**0.294)	Model: F1 score, AUC-PR statistic, Cohen’s Kappa statistic Feature: Chi-squared statistic
Rezapour et al. (2020)	Random Forest Support vector machine Multivariate adaptive regression splines Binary logistic regression	Fatal, incapacitating Injury Non incapacitating injury Possible injury Property damage only	Yes	**Temporal characteristic—**Posted speed limit, Irate other party, AADT, AADTT **Environment characteristic—**Operating speed, traffic volume, truck traffic volume, riding under the influence **Road characteristic—**horizontal curvature, wide roadway **Driver characteristic—**rider's age, RUI of alcohol, RUI of drug	Speed, traffic volume, truck traffic volume, riding under the influence, horizontal curvature, wide roadway, rider's age	RF-misclassification Rate 10% AUC**—**0.86 SVM-misclassification Rate 23% AUC**—**0.71 MARS-misclassification Rate 25% AUC**—**0.7 BLR-misclassification Rate 23% AUC**—**0.73	Model: Confusion matrix, AUC statistic Feature: RFE algorithm
Wahab and Jiang (2020)	Multi-layer perceptron Rule induction Classification and regression trees (SimpleCart)	Fatal, Hospitalized, Injured Damage	No	**Crash characteristic—**location type, time of the collision, collision type, collision partner **Road characteristic—**road description, settlement type, traffic control **Environment condition—**weather condition, time of the crash, the day of the week	Location type, settlement type, time of the crash, collision type and collision partner	Simple cart model**—**73.81% accuracy PART model—73.45% accuracy MLP model—72.16% accuracy	classification accuracy, precision, recall, TPR, FPR, AUC
Assi et al. (2020)	Feed-forward neural networks Support vector machine Fuzzy C-means clustering based feed-forward neural network Fuzzy c-means based support vector machine	Severe crashes, non-severe crashes	No	**Vehicle characteristics—**number of vehicles involved, vehicle type **Road characteristics—**road type, junction type, junction control **Environment characteristics—**light, weather, road surface condition, area type, speed limit, road class, number of causalities, day of the week	Vehicle attributes: Number of vehicles involved, Vehicle type Road condition: Road type, Road surface condition, Road class	SVM—73% accuracy FNN-FCM**—**71.8% accuracy SVM-FCM**—**74.2% accuracy	Accuracy, sensitivity, precision, F1 score
Arteaga et al. (2020)	Artificial neural Network support vector machine Naïve Bayes XGBoost Random Forest	–	–	–	–	GCV-LIME and LR**—**82.2% accuracy, GCV-LIME and LR-L1—28.9% accuracy, GCV-LIME and RF**—**46.7% accuracy GCV-LIME and XGBoost**—**62.2% accuracy LR-L1 and LR—51.5% accuracy LR-L1 and RF—42.4% accuracy LR-L1 and XGBoost—42.4% accuracy RF and LR—75.0% accuracy RF and XGBoost—45.8% accuracy LR and XGBoost—48.6% accuracy	Precision, recall, accuracy
Wali et al. (2021)	Text mining approach Ordered probit	Minor Major Fatal injury	No	–	–	–	–
Guo et al. (2021)	XGBoost	Property damage only, injury Fatal	No	**Vehicle characteristic—**vehicle movement, vehicle type **Driver characteristic—**driver move, driver factor, driver belt, driver sex **Older pedestrian characteristics—**older sex, older factor, older condition	Driver characteristic (alcohol, physical disability), older pedestrian characteristics, and vehicle movement	XGBoost—80.35% accuracy	ROC curve statistics
Mondal et al. (2020)	Random forest Bayesian additive regression trees	Severe Non-severe	No	**Crash condition—**manner of crash and type of intersection **Road condition—**route class, road surface condition **Environment condition—**weather conditions, month, work zone, day of the work, hour of the day, school bus, Light condition,	Manner of crash and weather conditions	RF**—**73% accuracy BART—61% accuracy	% IncMSE, R^2^ value
Fiorentini and Losa (2020)	Random tree K nearest neighbor Logistic regression Random forest	Property damage only, injury Fatal	No	**Environment condition—**number of vehicles, speed limit, light/weather condition, Day of the week, Number of causalities **Road condition—**junction detail, road surface condition, junction control, road type, urban or rural area	Day of the week, the number of casualties, the first road class, and the number of vehicles	RT**—**78.78% accuracy KNN**—**78.53% accuracy LR**—**85.74% accuracy RF**—**83.38% accuracy	Sensitivity, specificity, accuracy Recall, precision F1-score
Sarkar et al. (2020)	Support vector machine Artificial neural network Naïve Bayes k-nearest Neighbor Classification and regression tree analysis Random forest	Fatal Medical case, first-aid	No	–	–	–	Recall, F1-score and geometric mean
Kitali et al. (2021)	Firefly algorithm Support vector machine	Property damage only, injury, fatality	No	Driver condition—driver age Road condition—roadway surface condition, annual average daily traffic (AADT), type of separator, roadway terrain, left shoulder width, and right shoulder width, number of vehicles Environment condition**—**lighting condition, crash type, time of day, day of the week	Time of day, day of the week, roadway surface condition, and lighting condition, annual average daily traffic (AADT), type of separator, roadway terrain, left shoulder width, and right shoulder width, number of vehicles	FS-SVM Accuracy 61.90% Sensitivity 75.24% Specificity 37.52% F-score 71.86% AUC 0.57	Sensitivity, specificity, accuracy, recall, F1-score, AUC statistic

Researchers have employed several approaches to identifying the responsible factors, including econometric models, machine learning, and data mining frameworks. Applying traditional statistical and econometric models remains a workhorse in existing safety literature to identify the relevant features of crash risk and crash severity. Specifically, researchers have employed multinomial logit/probit, ordered logit/probit, count regression techniques, and generalized forms as random parameters and models for systematic heterogeneity aspects^10–12^.

With the emergence of advanced computing power, safety researchers have recently focused on the applications of machine learning approaches as an alternative analytical approach to modelling crash risk and severity. Several approaches were adopted using Artificial Neural Network^13^, Support Vector Machine^14^, and Logistic Regression^15^. Previous studies employed Decision Tree algorithms to analyze crash severity^10,13,14^. Advanced versions of Decision Tree such as Random Forest^16^, C4.5 algorithm^17^, Classification and Regression Tree^9^, and Multivariate Adaptive Regression Splines^1^ have also been identified to be adopted by several researchers.

Nevertheless, some past studies revealed that machine learning models have limitations in observing the correlation between the input and responsible variables. These studies also pointed out the limited concern on the feature selection process resulted in poor accuracy in crash severity prediction with the machine learning algorithm. Therefore, Pillajo-Quijia et al.^9^ emphasized the importance of feature selection and concluded that feature selection might improve the accuracy of crash severity prediction with machine learning algorithms. Rezapour et al.^17^ added that feature elimination could improve the accuracy of several machine learning algorithms, including Random Forest and Support Vector Machine. Inspired by these studies, Ghandour et al.^18^ implemented a feature selection technique using chi-square, which has significant importance in improving the accuracy of machine learning classifiers.

Several past studies implemented Logistic Regression^16–21^, Random Forest^8,9,16,19–21^, Classification Tree^1^ and C4.5 algorithm^21^ for crash severity prediction in different severity levels (i.e., serious or fatal/accident or non-accident/ possible injury or property damage, no-injury or minor injury or severe injury). These studies identified several influential factors, including driver characteristics (i.e., gender, age, residency, speed limit compliance, driver conditions); environmental characteristics (i.e., weather conditions, road conditions, and lighting conditions); roadway characteristics (i.e., roadway surface condition, crash location include vertical and horizontal characteristics of the segment and the posted speed limit of the segment). Fiorentini and Losa^19^ showed that the Logistic Regression model performs better in predicting property damage and fatal crashes with 85.74% accuracy. Similarly, Mafi et al.^21^ added that the Random Forest model performs well in no-injury, minor injury, or severe injury prediction with 87.15% accuracy. Rezapour et al.^1^ and Mafi et al.^21^ implemented the Classification Tree and C4.5 model and extracted 70% and 75.7% accuracy, respectively^17^.

Some studies adopted other machine learning models, namely Support Vector Machine^1,9,13,22^, k-nearest neighbor^19,23^, and Naïve Bayes^18,24^ to analyze a wide range of crash factors such as vehicle characteristics (i.e., vehicle age, condition of vehicle, weight of the vehicle, group of light trucks and vans, occupants involved, the body type of the vehicle), road infrastructure (road function, lane width, shoulder type, accident location), environmental condition (i.e., sight distance, lighting condition, weather, crash time, month/season) and temporal characteristics (i.e., crash type, time of day, day of the week, annual average daily traffic, type of separator, roadway terrain, left shoulder width, and right shoulder width, number of vehicles). Delen et al.^13^ revealed that Support Vector Machine performs better in low or high injury clarification and prediction with 90.41% accuracy. Fiorentini and Losa^19^ added that the k-nearest neighbors algorithm performs well in identifying the fatal injury and property damage with 78.53% prediction accuracy.

Several studies investigated the impacts of several factors by using XGBoost, Artificial Neural Network/Feed-forward Neural Networks/Multi-layer perceptron, and Mixed Logit model to predict crash severity and injury severity. Guo et al.^25^ suggested that XGBoost algorithm performance prediction is higher than the Artificial Neural Network/Feed-forward Neural Networks algorithm with 80.35% accuracy. Uddin and Huynh^26^ revealed that the Mixed Logit model performs better than other machine learning models with 99% accuracy in crash analysis and prediction. Wahab and Jiang^14^ and Assi et al.^15^ concluded that the multi-layer perceptron model performs better than the feed-forward neural networks model with 72.16% and 71.8% accuracy, respectively.

Additionally, few researchers employed Classification and Regression Tree, Bayesian additive regression trees, and the Simple Cart model to investigate additional responsible factors. Pillajo-Quijia et al.^11^ and Mondal et al.^10^ developed a model based on the Classification and Regression Trees and Bayesian Additive Regression Trees. They predicted crash severity based on weekdays, months, type of road surface construction, and lane width with 78% and 61% accuracy, respectively. Wahab and Jiang^14^ developed a model based on the Projective Adaptive Resonance Theory and Simple Cart algorithm model. The study found that Simple Cart performs better than other statistical models. While the previous studies performed well with regard to traditional machine learning models, few of these studies focused on the feature selection process in crash severity analysis and prediction^1,26^.

Hence the significant outcome of the discussed works is noticeable. It is noteworthy to mention that it has already been argued in several past studies that the feature selection process improves the classification and prediction of models in the context of crash severity analysis. Without a feature selection step, the model may lead to poor results with inappropriate predictions and result in inappropriate countermeasures specific to the crash-responsible factors. Following this, a few studies implemented several feature selection techniques to improve the crash severity analysis in terms of classification and prediction results^1,11,17,18^. However, these studies relied on a single feature selection technique/algorithm, while considering multiple feature selection techniques might alter the final result. Moreover, the majority of the existing studies consider the official databases that are freely accessible to the research community, though an assorted number of issues associated with unreported data are observed. Therefore, an alternative source of official database is an emerging need in this current era. In these drawbacks, the significance of the proposed approach is aligned with the analysis of the crash data compiled from newspaper data as an alternative source of official data considering multiple feature selection techniques and machine learning algorithms to represent the crash severity statistics and identify the associated risk factors that are responsible for serious crashes in developing countries like Bangladesh.

## Study design

### Data gathering

**Figure 1:**
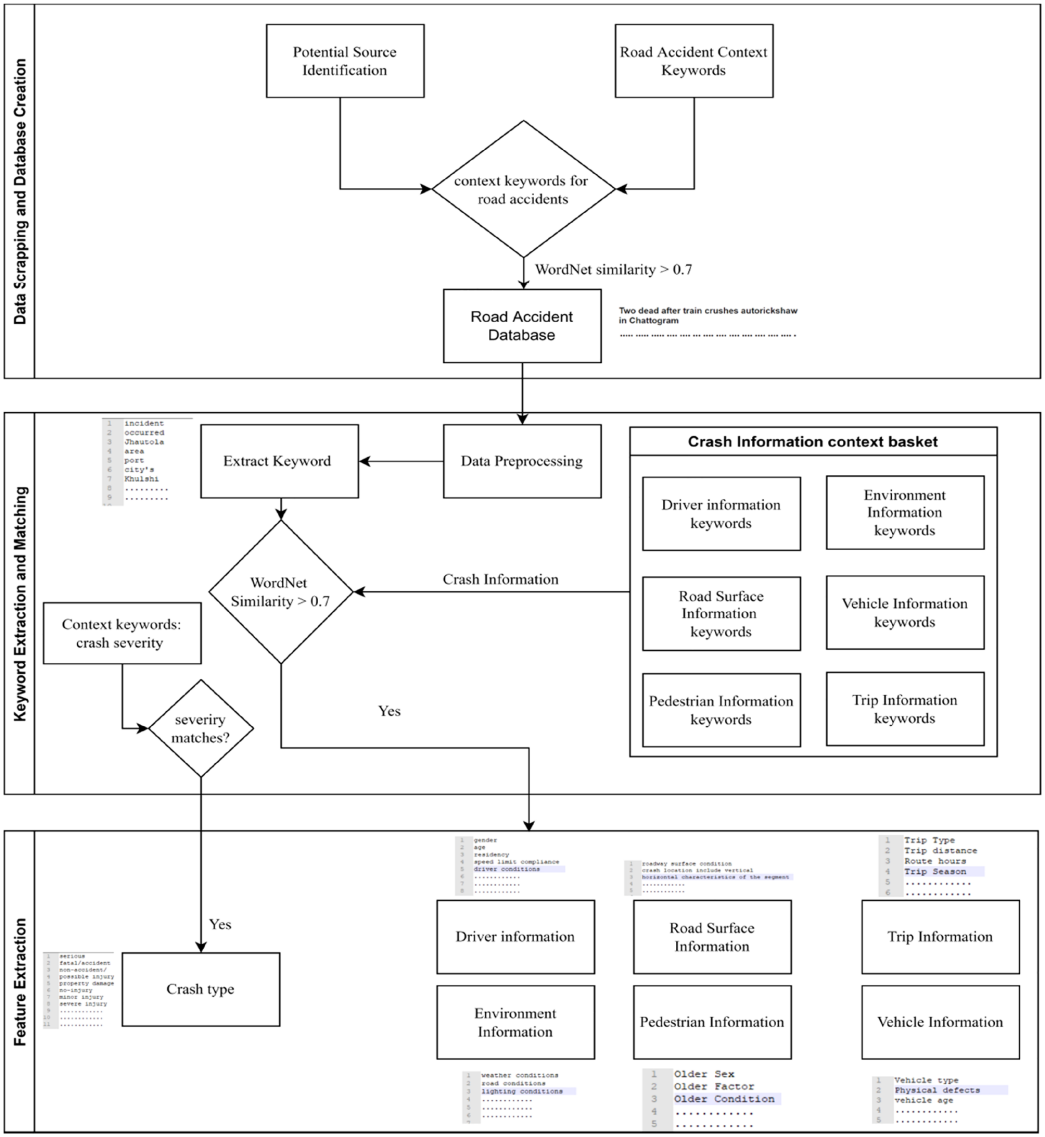
Overview of crash data collection from daily newspaper articles.

The major focus of this study is to develop and propose a data-scrapping algorithm for developing a compiled crash database from different Newspaper articles. In this study, we have compiled crash records reported in several newspapers of Bangladesh for the year 2019. We opted for three newspapers: Daily Prothom Alo (printed and e-paper), Daily Jugantor (printed and e-paper), and Bdnews24 (e-paper/online portal) according to their popularity, daily circulation number, and readers on e-portal. As of May 2021, the Daily Prothom Alo, and the Daily Jugantor are the oldest newspapers in Bangladesh, and their circulation numbers are 501,800 and 290,200, respectively. Besides, according to the statistics of July 2015, the number of readers on the Bdnews24 e-portal is 6.6 million. Therefore, these three newspapers are the most circulated and popular newspaper that covers a wide range of news, including crash reports which led us to consider these three newspapers for compiling the crash data in this study. The proposed approach can further be augmented by compiling crash information from other newspapers. The crash data compilation algorithm and process from the daily newspaper are presented in Fig. 1 and are discussed in the following sections.

#### Tier 1—data scraping and database creation

To create a Bangladesh road crash database, we first searched several potential sources of data where the crash reports were published. We selected three top newspapers to identify the road crash context data. For that, we created a context keyword set for road crashes where different words were stored to match the news keywords, such as road accident and road crash. After finding the semantic similarity (WordNet similarity), the news article was included in the road crash database if the similarity value was greater than the threshold value of 0.7.

#### Tier 2—keyword extraction and matching

Data preprocessing was an important part of keyword extraction to omitted words like 'the', 'as', and 'are'. After completing the preprocessing steps (data cleaning, integration, transformation, reduction, and transformation), we extracted keywords from the preprocessed text, such as “occurred” and “accident”. In the meantime, a road crash information context basket was created to find the semantic similarity between the extracted keywords and the basket words. For instance, the driver's information basket contains the driver’s age and gender information. The vehicle information basket includes vehicle age and type-related information. If the keyword (WordNet similarity value) is greater than the threshold value, then the keyword was used for tier 3. In parallel, we evaluated the severity level by using a context keyword set of severity levels.

#### Tier 3—features extraction for final dataset

The features were selected based on the output of tier 2. We used the crash type and responsible factors of crash severity (Fig. 1) as the key features to create the final dataset for the proposed model.

Based on the three major Newspaper articles, the newspaper archived crash database collected for the Year 2019 across Bangladesh contains 441 crash records while also reporting information relevant to vehicle characteristics, environmental characteristics, driver characteristics, road characteristics, and residential location characteristics. The identified features are summarized in Table 2. The crash severity was reported in the database as a three-point severity scale variable: non-fatal injury, severe injury, and extremely severe injury. From Table 2, it could be observed that, among 441 records, 2.26% of crashes were non-fatal, 73.92% were severe, and 23.80% were extremely severe injuries.

### Data pre-processing

Data preprocessing is an essential element of data analysis to assure the quality and reliability of the result. Therefore, we performed some data preprocessing before implementing the model. The preprocessing was performed by removing duplicate records and eliminating unexpected notations. After completing the preprocessing, fifteen features were initially selected for further analysis. Fourteen features were considered as input attributes, and crash severity outcome was considered as the output of this study. Crash severity is defined as three-point variables: non-fatal crash (if there were zero fatalities), severe crash (if one or two crash victims were fatally injured), and extremely severe crash (if three or more than three crash victims were fatally injured). Table 2 shows the input and output features with their detailed description.

**Table 2:** Summary of features included in this study.

Crash severity levels (no of observations = 441)	Non-fatal (%)		Severe (%)		Extremely severe (%)		
	2.26%		73.92%		23.80%		

Features	Type	Categories	Description	Features	Type	Categories	Description
**Input features**							
Vehicle type	Nominal	10	Motorcycle Tractor, Car Auto Rickshaw, Footpath Bus, Truck MicroBus, BiCycle Mini-Truck	Weather	Nominal	4	Sunny Gloomy Rainy Hot
Time of crash	Nominal	2	Day, night	Lighting	Nominal	3	Dark Light Grey
Day of crash	Numerical	12	Day**—**1–31; month**—**12: January, February March, April, May June, July, August September, October, November, December	Driver age	Nominal	4	Teenager (ages < 18 years) Young adults (ages 18–35 years) Middle-aged adults (ages 36–55 years) Older adults (ages > 55 years)
Gender	Nominal	2	M**—**male, F**—**female	License Type	Nominal	2	P—Professional NP—Nonprofessional
Road classification	Nominal	6	NH**—**National Highway RH**—**Regional Highway ZR—Zila Roads UpR—Upazila Road UnR—Union Roads VR—Village Roads	Seat belts	Nominal	2	Y—Yes N—No
Number of lanes	Numeric	6	NH = 6 RH = 4 ZR = 2 UpR = 1 UnR = 1 VR = 1	Vehicle Age	Numeric	3	10 years 20 years 30 years
Road Surface Type	Nominal	5	Diamond Interchange Zig Zag Road J Turns Circular Road Normal				
Residential Location	Nominal	75	Jhenaidah, Gopalganj, Fouzdarhat, Chattogram, Thakurgaon, Barisal, Bogra, Dhaka, Rajbari, Narayanganj, Cox's Bazar, Jessore, Hobiganj, Naogaon, Netrokona, Sherpur, Rajshahi, Kushtia, Madaripur, Munshiganj, Bagerhat, Jaypurhat, Jatrabari, Faridpur, Sreenagar, Comilla, Noakhali, Satkhira, Gaibandha, Shariatpur, Tangail, Rangpur, Panchagar, Lakshmipur, Shatkhira, Pabna, MoulviBazar, Patuakhali, Khulna, Gazipur, Natore, Sirajganj, Magura, Pirojpur, Dinajpur, Nilphamari, Feni, Bhola, Sylhet, Kishoreganj, Chuadanga, Ashulia, Bandarban, Rahobol , Kurigram, Narsingdi, Mymensingh, Wazirpur, Hathazari, Brahmanbaria, Godagari, Siddhirganj, Lalmonirhat, Sunamganj, Chadpur, Manikganj, Chapainnawabganj, Savar, Jamalpur, Rangamati, Gobindaganj, Hili, Rampal, Khagrachhari, Keraniganj				
**Output feature**							
Crash Type	Nominal	3	Severe Death ≥ 3 Medium 2 ≤ death ≤ 1 Mild Death = 0, Injured only *Medium—326, mild—10, severe**—**105				

### Analytical context of current study

**Figure 2:**
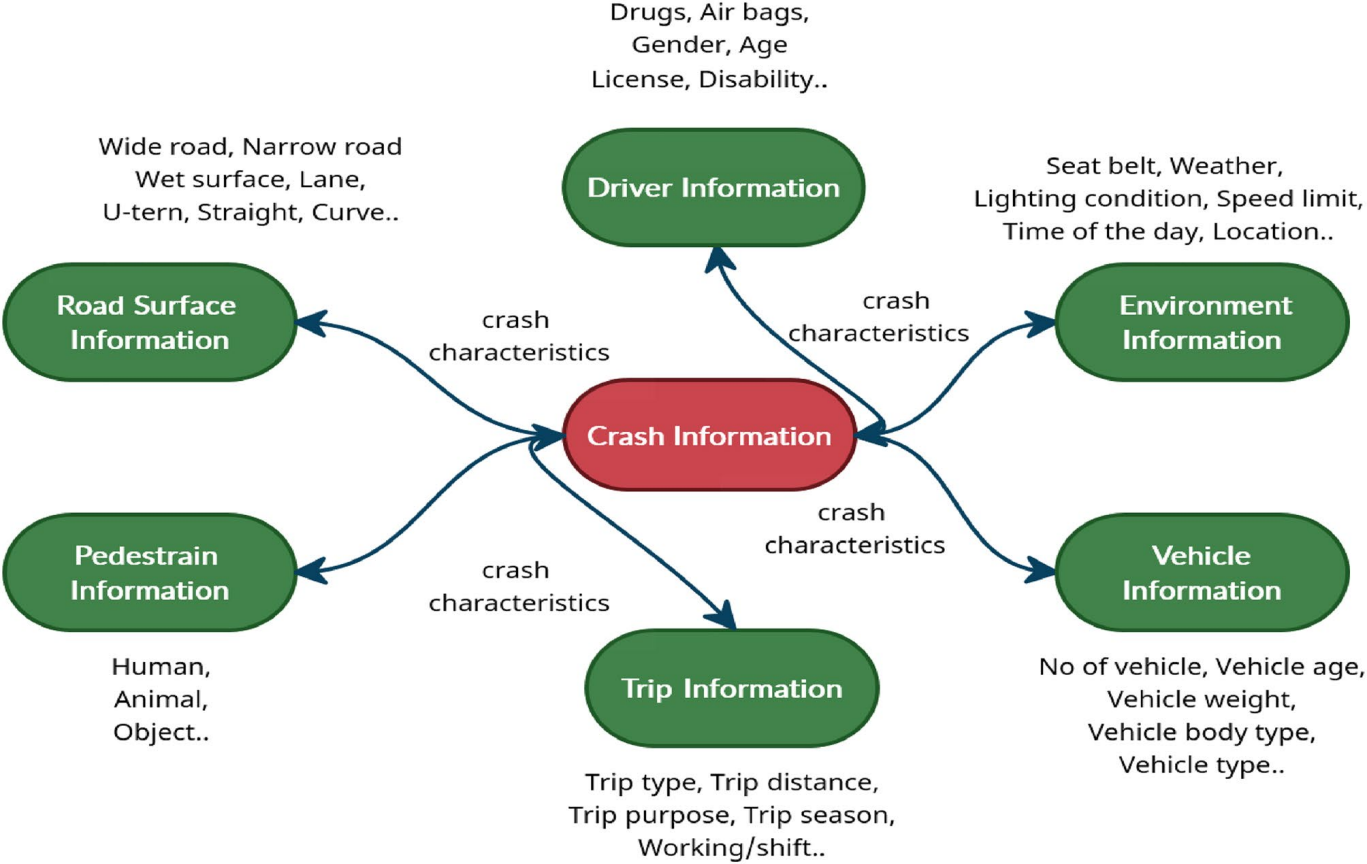
Responsible factors of crash severity.

The major focus of studies analyzing crash data in existing safety literature is identifying the responsible features of crash risk and crash severity outcomes. Our current study contributes towards the second stream of studies. Several studies examined both intrinsic and extrinsic factors related to crashes for crash severity analysis. These studies have identified a multitude of factors to be responsible for crash severity outcomes, including highway functional class, roadway geometry, demographics of road users, weather condition, and other situational attributes^15,17,23,25,27^. Several existing studies investigated the impacts of driver characteristics, vehicle characteristics, and pedestrian characteristics in understanding the crash severity mechanism^11,28^. A summary of these responsible factors of crash severity outcomes is illustrated in Fig. 2.

The prime objective of this study is to determine the importance of feature selection techniques in the crash severity prediction of Bangladesh by using the crash information collected from daily newspapers for the year 2019. Specifically, this study aims to apply machine learning-based algorithms to provide a detailed understanding of responsible factors for crash severity outcomes. Our preliminary observation from these studies is that the Decision Tree, Random Forest, and Naïve Bayes approaches are frequently applied machine learning algorithms in road safety research for their regularization parameter. These algorithms are important in avoiding over-fitting, better performance with imbalanced data, and non-linearity for input and target variables^22^. Among several feature selection techniques, chi-square, two-way ANOVA, and regression analysis have also been considered for their less complexity, effectiveness, and cost-sensitive properties. Therefore, in our crash severity analysis and prediction model, three machine learning techniques, namely Decision Tree, Random Forest, and Naïve Bayes, with three feature selection techniques (chi-square, Two-way ANOVA test, and Regression analysis) have been adopted for crash severity prediction. Generally, Decision Tree, Random Forest, and Naïve Bayes are traditional and popular algorithms in machine learning research. Delen et al.^13^ reported that Decision Tree provides better results compared to other models (such as the Probit model) introduced by Mondal et al.^10^. Similarly, some previous studies have implemented a Random Forest algorithm for crash severity analysis and prediction. These studies concluded that Random Forest performed better than other machine learning algorithms on both small and large datasets in crash severity identification. Beyond these traditional algorithms, we have also adopted the Naive Bayes technique, including Multinomial Naïve Bayes and Gaussian Naïve Bayes techniques. Naïve Bayes techniques are likely to be an effective classifier with a large amount of data in the automatic learning process. It is easy to implement and helps to improve the efficiency of the final result. The effectiveness of the proposed approach has been evaluated through precision, F1 score, classification accuracy, sensitivity, and specificity to understand the crash severity outcome and responsible factors.

## Crash severity analysis and responsible factors prediction

**Figure 3:**
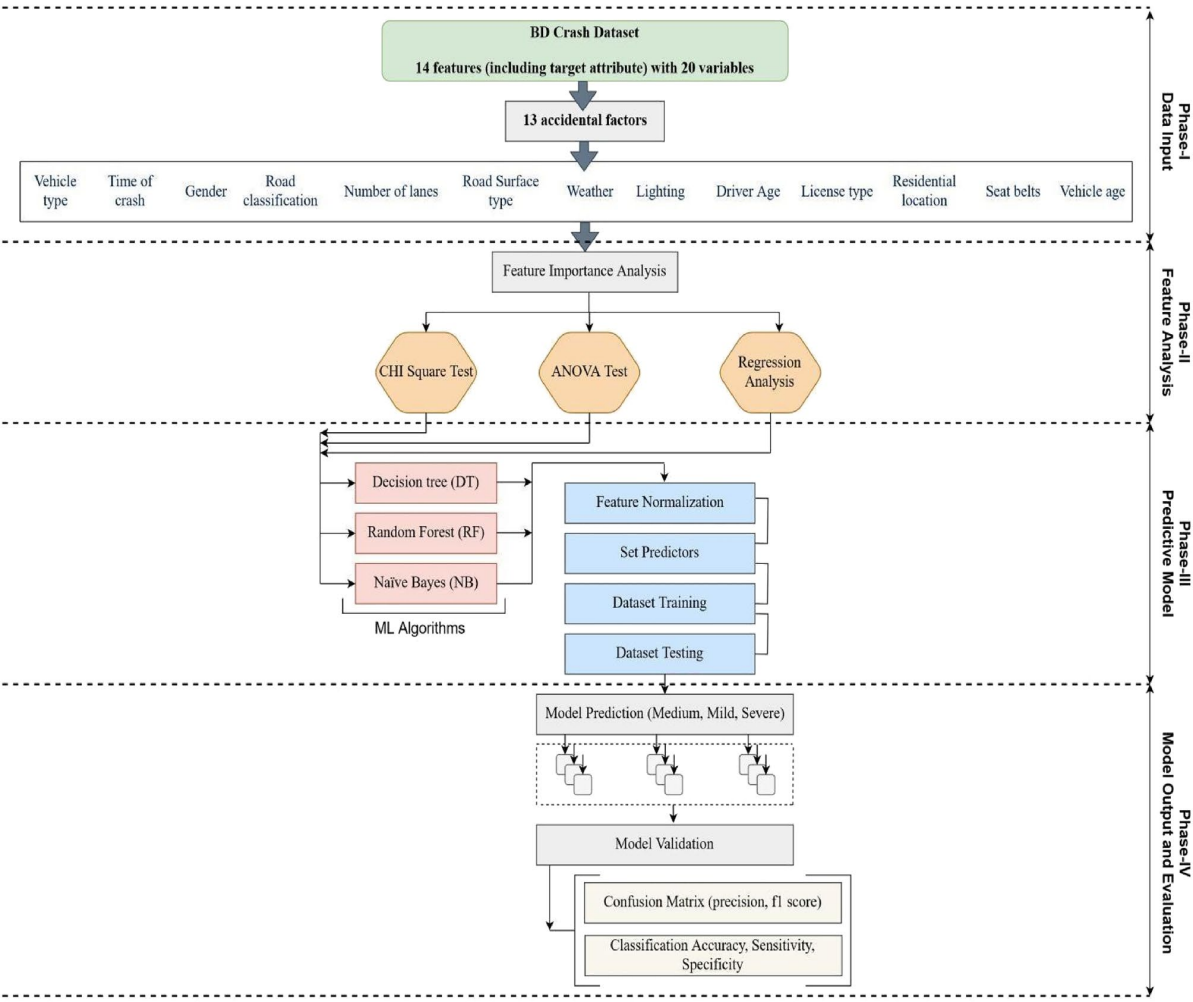
Analytical framework of crash severity analysis.

In this section, we present the crash severity prediction model to identify responsible factors that are related to crash severity outcomes. The workflow diagram of the proposed crash severity prediction model is illustrated in Fig. 3. The crash severity and prediction analysis were performed in four phases: data input (Phase-I), feature analysis (Phase-II), model specification (Phase-III), and model evaluation (Phase-IV). The detailed working process of the proposed model is discussed in the following subsections.

### Feature analysis approach

This section considers feature selection techniques (Fig. 3, Phase-II) to evaluate the input feature's importance in predicting the actual output (crash severity outcome). This study considered chi-square statistics, Two-way ANOVA statistics, and Regression analysis statistics to identify the responsible features for executing our proposed model. A brief explanation of feature selection statistics is given below.

#### Chi-square

Chi-square is a statistical measurement of two dependent and independent attributes. It is also known as the categorical feature evaluation technique, which provides the correlation information of two attributes to show the difference between two sets of data (observed and target dataset). Equation (1) shows the process of chi-square statistic calculation where "c" is the degree of freedom, "O" refers to the observed value and "E" refers to the expected value.

1 $${\text{X}}^{{2}} = \sum ({\text{Oi}}{-}{\text{ E}}_{{\text{i}}} )^{{2}} {\text{/Ei}}$$

**Figure 4:**
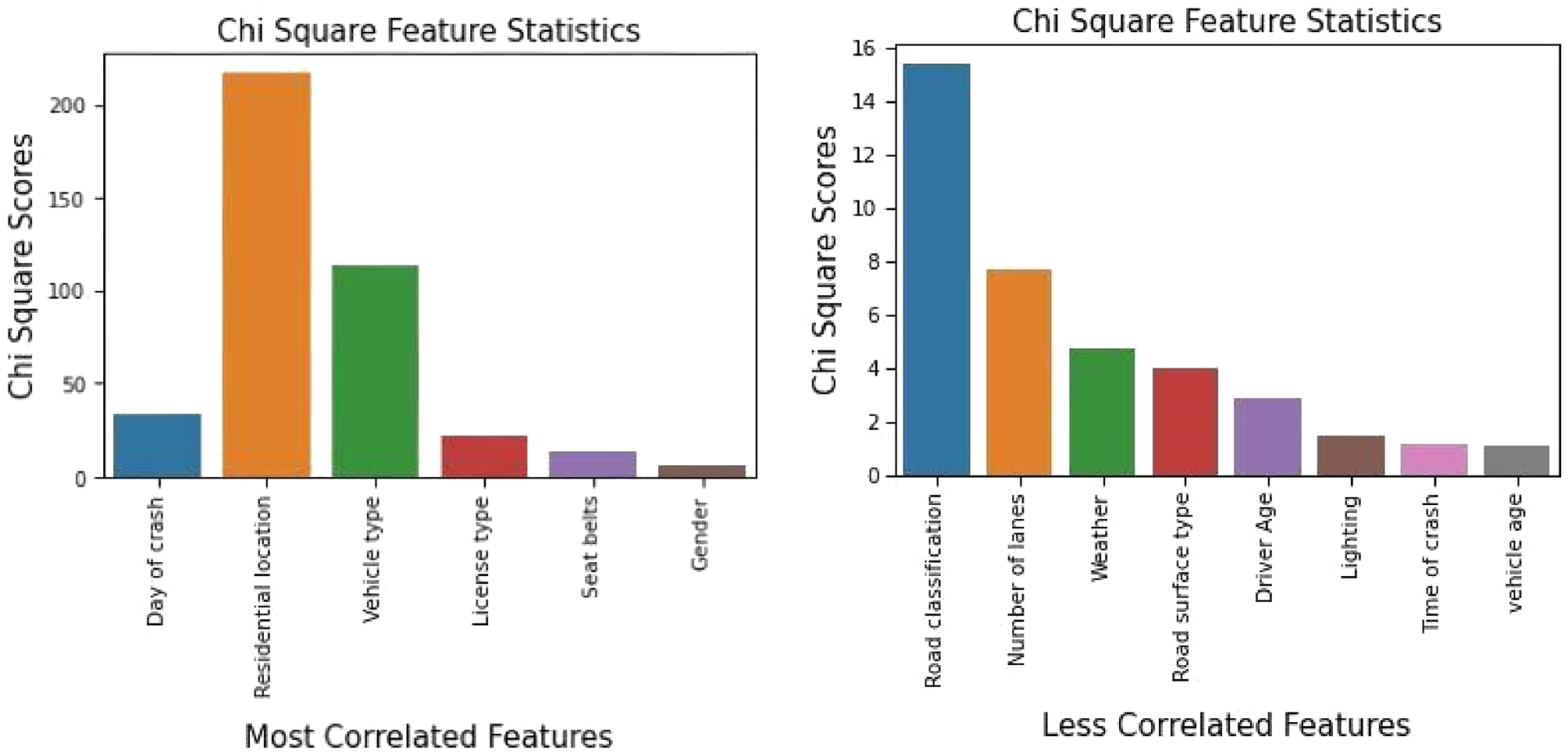
Chi-square feature statistics.

To evaluate the chi-square statistic, we consider the chi-square critical value under alpha level 0.05 (5%) and set our threshold value to α = 0.05 and found six features as the most correlated features such as Day of Crash, Residential Location, Vehicle Type, License Type, Seat Belts, and Gender. Figure 4 shows the graphical representation of the most correlated and less correlated features identified in the crash dataset with their corresponding chi-square scores.

#### Two-way ANOVA

We consider the Two-Way ANOVA Test statistic to identify the substantial impact in two groups of data (input and target). Two-way ANOVA helps determine whether the null hypothesis should be accepted or rejected.

**Figure 5:**
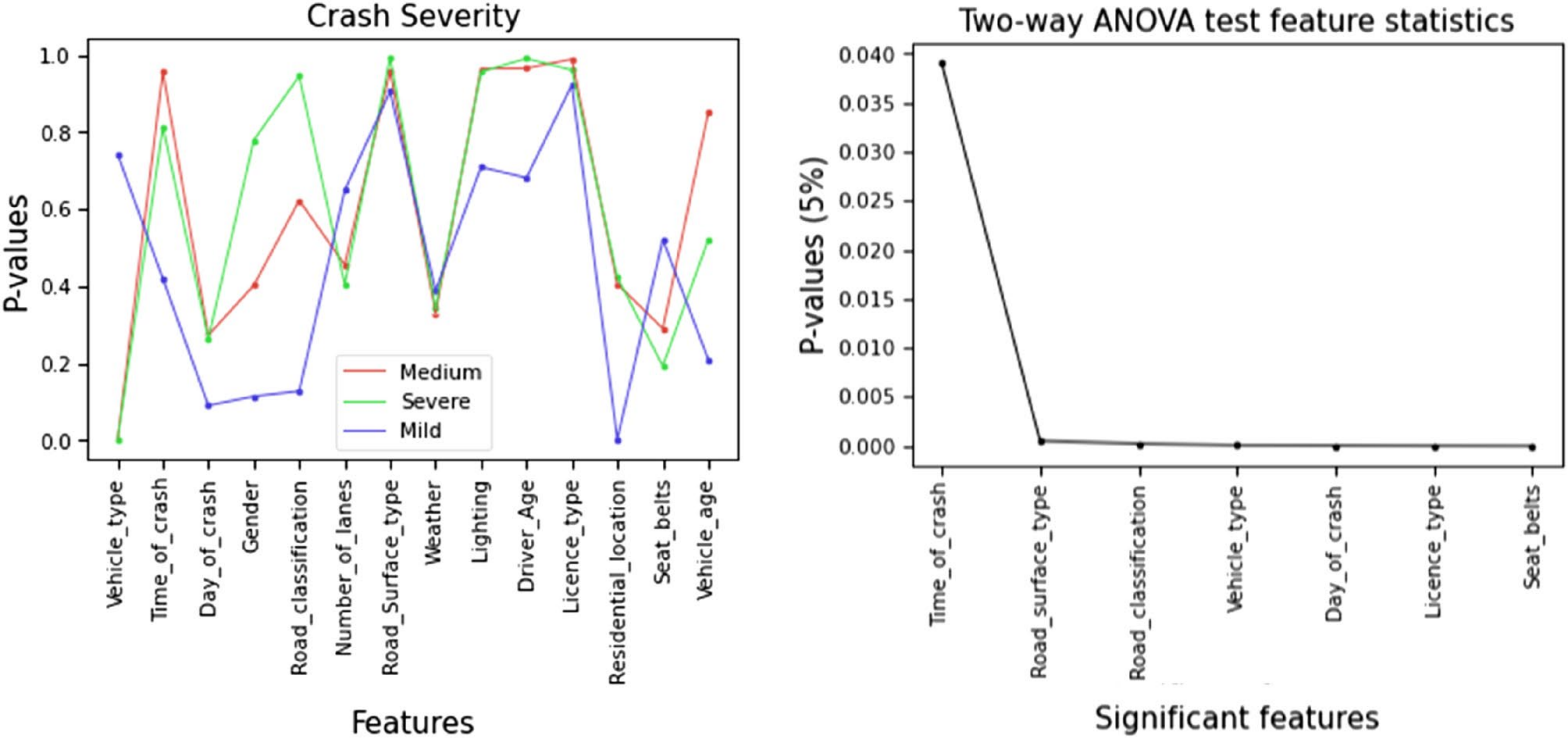
Two-way ANOVA test feature classification for significant feature identification.

To evaluate the null hypothesis, we consider p values. p value was classified through a significance level (ð) where, if the p value was greater than the significance level (p value > ð), then there was no significant impact between the two groups of data. Similarly, if the p value was less than the significance level (p value < ð), there was a significant impact between the two data groups. We set the significance level ð = 0.05 and found seven significant features: Vehicle Type, Day of Crash, License Type, Road Surface Type, Road Classification, Seat Belts, and Time of Crash. Figure 5 shows the Two-way ANOVA test result of significant features with their p value from the fourteen input features.

#### Regression analysis

**Figure 6:**
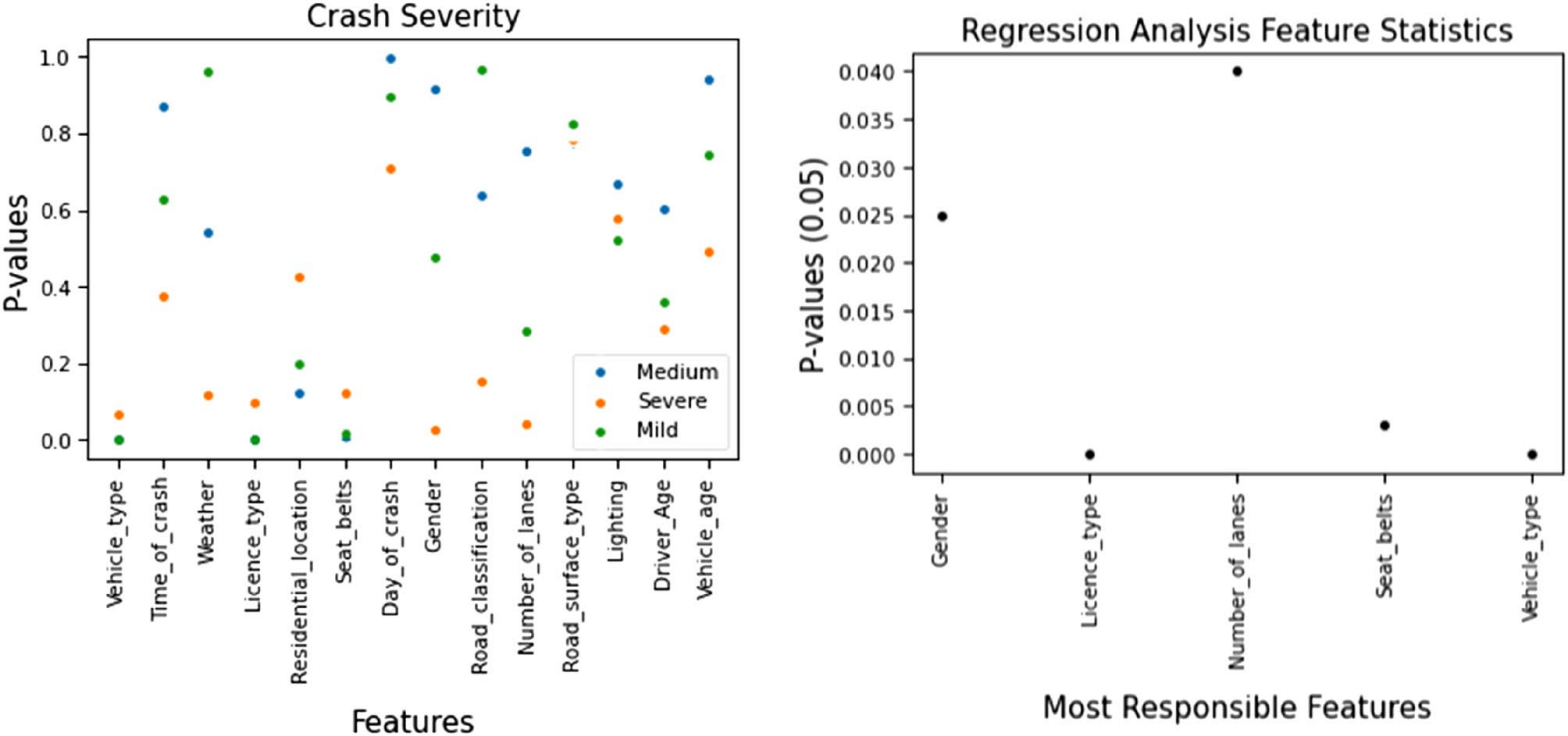
Regression analysis feature statistics to identify the most responsible features.

Regression analysis is a statistical method to evaluate the features and understand the impact of each input feature on the output feature. It helps to determine the features that we should consider. Besides, it also helped get the desired output or better model performance. Therefore, through regression analysis, our prime goal was to identify the most important features or factors of data. To evaluate the importance of input features through Regression analysis, we consider the p value. To evaluate p values, a threshold value was set to α = 0.05 and found five features responsible for crashes: Gender, License Type, Number of Lanes, Seat Belts, and Vehicle Type. Figure 6 shows the responsible feature identification of the BD crash dataset through regression analysis.

### Model specification

This section describes several machine learning classifiers or predictive models (Fig. 3, Phase-III) that are effective in handling crash severity models. In this study, three machine learning models were considered: Decision Tree, Random Forest, and Naïve Bayes (Multinomial and Gaussian Naïve Bayes). These machine learning models had been implemented using the Python framework through a collaboratory environment with Python data learning libraries, such as Pandas and scikit-learn. As in our crash dataset, all the features are categorical, so encoding categorical data into numerical data is crucial. Therefore, we implemented a popular and effective encoder, namely Label Encoder, through the Python Sklearn library. Generally, Label Encoder generates a non-repeat numerical value for each feature of the data^12^. After data encoding, our selective machine learning classifiers were implemented for crash severity prediction. A brief discussion about the machine learning classifiers used in this study has been described in the following.

#### Decision Tree (DT)

Since the early 1930s, Decision Tree has been popular and effective for various decision-making problems using expert knowledge^18^. In recent days, it has become a popular and effective analytics tool in data mining over other machine learning techniques (i.e., neural network or support vector machine). Decision Tree is a supervised machine learning classifier that works by splitting categorical data to create a model that predicts the value of a target attribute. Therefore, in our model, we used Decision Tree to analyze our categorical features to predict several crash severity outcomes.

#### Random Forest (RF)

Random Forest is a popular supervised machine learning classifier widely used in several classification and prediction applications. Random Forest machine learning classifiers work by constructing multiple decision trees of the given dataset during the learning phase and learning both the dataset's usual and unusual patterns during the training phase. It predicts high accuracy output for large and small datasets, even for missing data in the dataset. Previous research also added that Random Forest works much better than traditional decision trees. It combines the output of the entire decision tree and calculates their average to reduce overfitting and improve prediction accuracy.

#### Naïve Bayes (NB)

Naïve Bayes classifier is a widely used machine learning classifier that is easy to use for large-scale datasets and fast to predict in multi-class prediction. It is also known as a probabilistic classifier that performs based on Bayes' Theorem. Equation (2) shows the process of Naïve Bayes classifier prediction methods where "x" is the predictor and "c" is the prediction class or target class. Some specialized Naïve Bayes classifiers have recently been introduced for crash severity prediction. Therefore, we considered the two most advanced Naïve Bayes classifiers in this study, namely: Multinomial Naïve Bayes and Gaussian Naïve Bayes classifiers. Multinomial Naïve Bayes is a specialized version of Naïve Bayes classifier which automatically learns from the input features through an automated learning process. Also, it refers to the Multivariate Event Model, which is accurate for making predictions. Generally, it performs through multidimensional and polynomial probability theorems to overcome several data ambiguities. The multidimensional model was used to handle the missing attributes of the data and make a prediction. Besides, the polynomial model was used to record each attribute occurrence to identify the attributes that do not appear in the dataset. Gaussian Naïve Bayes classifier is another specialized version of Naïve Bayes classifier, which deals with multi-class data to predict according to Gaussian distribution parameters. It works by segmenting data according to each class and then computes each class's mean and variance to make predictions of input values (x) associated with observation values (v).

2 $$\mathrm{Pc}|\mathrm{x }=\mathrm{Px}|\mathrm{c P}(\mathrm{c})\mathrm{ P}(\mathrm{x})$$

Here, P (c|x) is the posterior probability of the target class, P (c) is the prior probability of the target class, P (x|c) is the probability of the predictor class, P (x) is the prior probability of the predictor class.

### K-fold cross-validation

Identifying the actual accuracy of a prediction model induced by a supervised learning algorithm is effective. It helps to estimate future prediction accuracy and choose a better estimating classifier. The more popular methodology for identifying the actual accuracy of the model is splitting the dataset for training and testing^13^. Some previous literature concludes that a conventional implementation of the dataset-splitting method is known as k-fold cross-validation. K-fold cross-validation is also called v-fold cross-validation. Generally, k-fold cross-validation is a process of splitting a complete dataset (all the rows/columns) into k-distinct subsets. To execute the experiment, first, k − 1 number of records trained into the desired classifier, and the remaining subset was used to test or validate the model. This experimental process repeated k-times with different folds and used each fold to test the classifier. Then the remaining data of the dataset was used for training purposes. Finally, the overall accuracy of the classifier was calculated using the average/mean calculation of the k number of test accuracy. Equation (3) shows the K-fold accuracy calculation process. In our study, we used tenfold cross-validation, where we considered the value of k = 10

3 $$\mathrm{Cross}-\mathrm{validation\ Accuracy }\left(\mathrm{CVA}\right)=\frac{1}{z} {\sum }_{i=1}^{k}Accuracy (i)$$

### Model evaluation

Several evaluation metrics were used for the performance measurement of machine learning models. Therefore, to understand and evaluate the performance of our machine learning-based crash severity prediction model, here, we used a confusion matrix for both testing and training output data. A confusion matrix was used to evaluate the performance of our prediction model. It also helps to identify the actual value of our test data. The confusion matrix was beneficial to calculate several other performance measurement techniques, namely classification accuracy, precision, sensitivity, specificity, and F1 Score (Fig. 2, Phase-IV), which shows in Eqs. (4), (5), (6), (7), and (8), respectively.

Here, classification accuracy refers to the ratio of the number of correctly predicted crashes and the total number of observed crashes. Classification accuracy is defined in Eq. (4):

4 $$Classification\ Accuracy=\frac{Number\ of\ correctly\ predicted\ crashes \left(TP+TN\right)}{Total\ number\ of\ crashes (TP+TN+FP+FN)}$$

Here, TP = number of true positive crashes, TN = number of true negative crashes, FP = number of false positive crashes, FN = number of false negative crashes.

Furthermore, we consider other evaluation matrices such as precision, recall/sensitivity, specificity, and F1 score. Precision is the ratio of correctly predicted crashes as a non-fatal, severe, or extremely severe injury outcome to the total number of correctly predicted crashes as non-fatal, severe, or extremely severe. The precision calculation of non-fatal injury of the crash severity prediction model is shown in Eq. (5). The recall is also known as sensitivity which can be defined as the ratio of correctly predicted crashes as non-fatal, severe, or extremely severe to the total number of actual non-fatal, severe, or extremely severe crashes. Equation (6) represents the process of sensitivity analysis of non-fatal injury.

5 $$Precision \left(i, j, k\right)=\frac{Number\ of\ correctly\ predicted\ non-fatal\ crashes\left(TP\right)}{Total\ number\ of\ correctly\ predicted\ non-fatal\ crashes (TP+FP)}$$

6 $$Recall/sensitivity \left(i, j, k\right)=\frac{Number\ of\ correctly\ predicted\ non-fatal\ crashes\left(TP\right)}{Total\ number\ of\ actual\ non-fatal\ crashes (TP+FN)}$$

Several studies suggested that a high value of precision and recall could predict the best prediction classifier^14^. But, extracting high precision and recall is difficult and sometimes impossible^15^. Therefore, to minimize this problem, the concept of F1 score was introduced that could predict the model's performance more perfectly and be accepted by the researcher. Generally, the F1 score is the harmonic mean of precision and recall, which define through Eq. (7). Furthermore, we also consider the specificity. Specificity is the process of identifying the crashes that are negatively classified as non-fatal, severe, or extremely severe injury crashes. It is the ratio of predicted crashes among the total number of actual predicted crashes as non-fatal, severe, or extremely severe outcomes. The specificity calculation process for non-fatal injury is defined through Eq. (8).

7 $$F1 score \left(i, j, k\right)=\frac{2*Precision*Recall}{Precision+Recall}$$

8 $$Specificity \left(i, j, k\right)=\frac{Number\ of\ predicted\ non-fatal\ crashes\left(TN\right)}{Total\ number\ of\ actual\ non-fatal\ crashes (TN+FP)}$$

## Evaluation result

To evaluate the performance of three machine learning classifiers with different feature selection techniques in crash severity prediction, in this study, we performed the evaluation through two evaluation criteria: first, considering precision and F1 score, and second, through classification accuracy, sensitivity, and specificity. The experimental result with a detailed discussion is described in the following.

### Evaluation-1

To identify the best classifier for the crash severity prediction model, precision and F1 score are effective and recommended by previous researchers. Therefore, this study evaluated our three prediction classifiers (Decision Tree, Random Forest, and Naïve Bayes) results through precision and F1 score statistics. We conducted five tests for each classifier with three feature selection processes: chi-square, Two-way ANOVA test, Regression analysis, chi-square ∪ Two-way ANOVA ∪ Regression analysis, and chi-square ∩ Two-way ANOVA ∩ Regression analysis.

**Figure 7:**
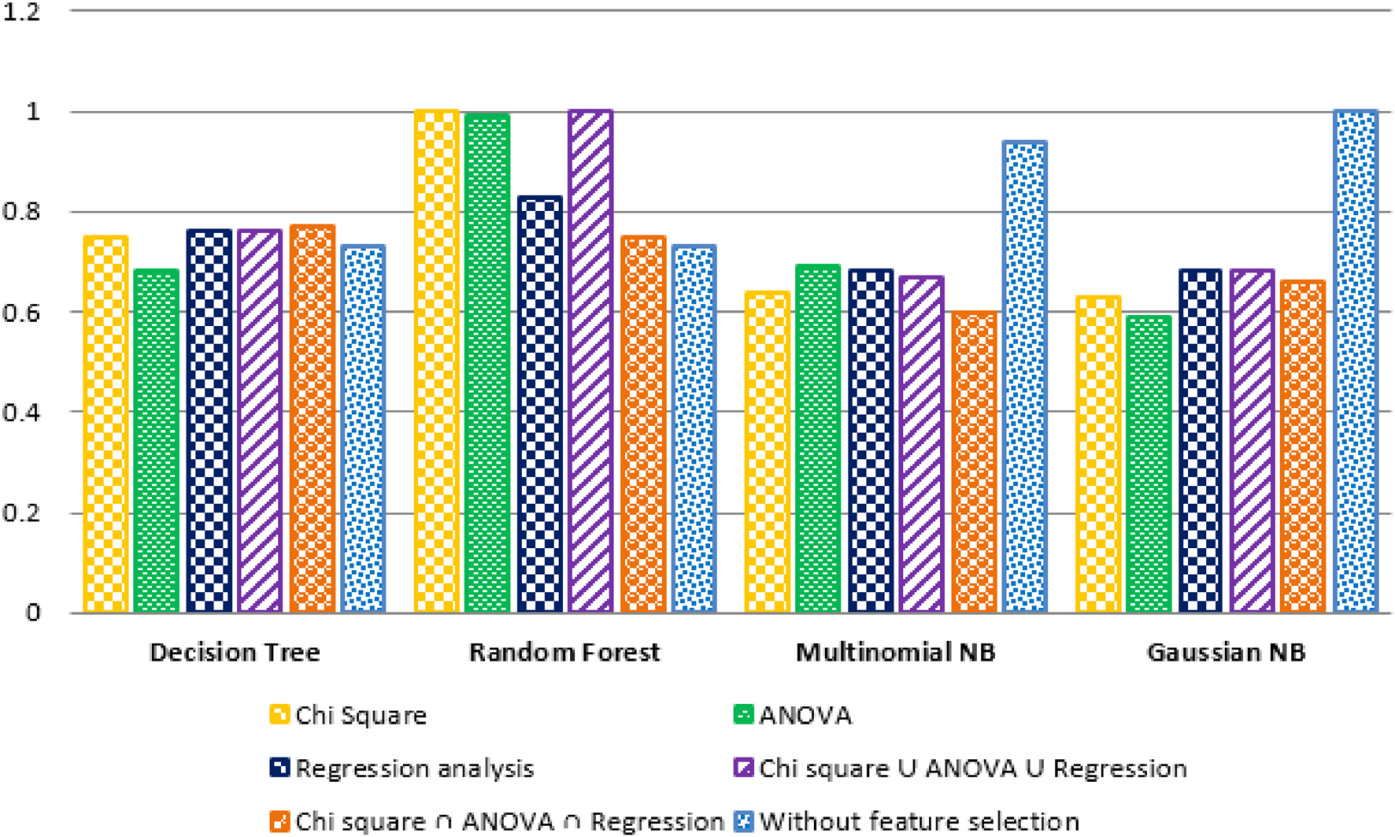
Comparative result of different classifiers considering precision score (feature selection and non-feature selection).

Our prime goal was to identify the effective machine learning classifier for crash severity prediction in this study. Therefore, we evaluated the prediction result through each classifier's weighted average precision measure and weighted average F1 score, considering both feature selection and non-feature selection processes. First, Fig. 7 shows the graphical representation of the experimental result based on the weighted average precision (y-axis) of three classifiers: Decision Tree, Random Forest, and Naïve Bayes (Multinomial and Gaussian) (x-axis). It shows that Random Forest gives the better result for all the feature selection techniques, specifically, providing the high accuracy for {chi-square} and {chi-square ∪ Two-way ANOVA ∪ Regression} with a maximum of 1.0 weighted average precision. Besides, the Decision Tree had the best result for {chi-square ∩ Two-way ANOVA ∩ Regression} feature selection technique with a maximum of 0.77 weighted average precision. In contrast, Multinomial Naïve Bayes and Gaussian Naïve Bayes were found effective without a feature selection process.

**Figure 8:**
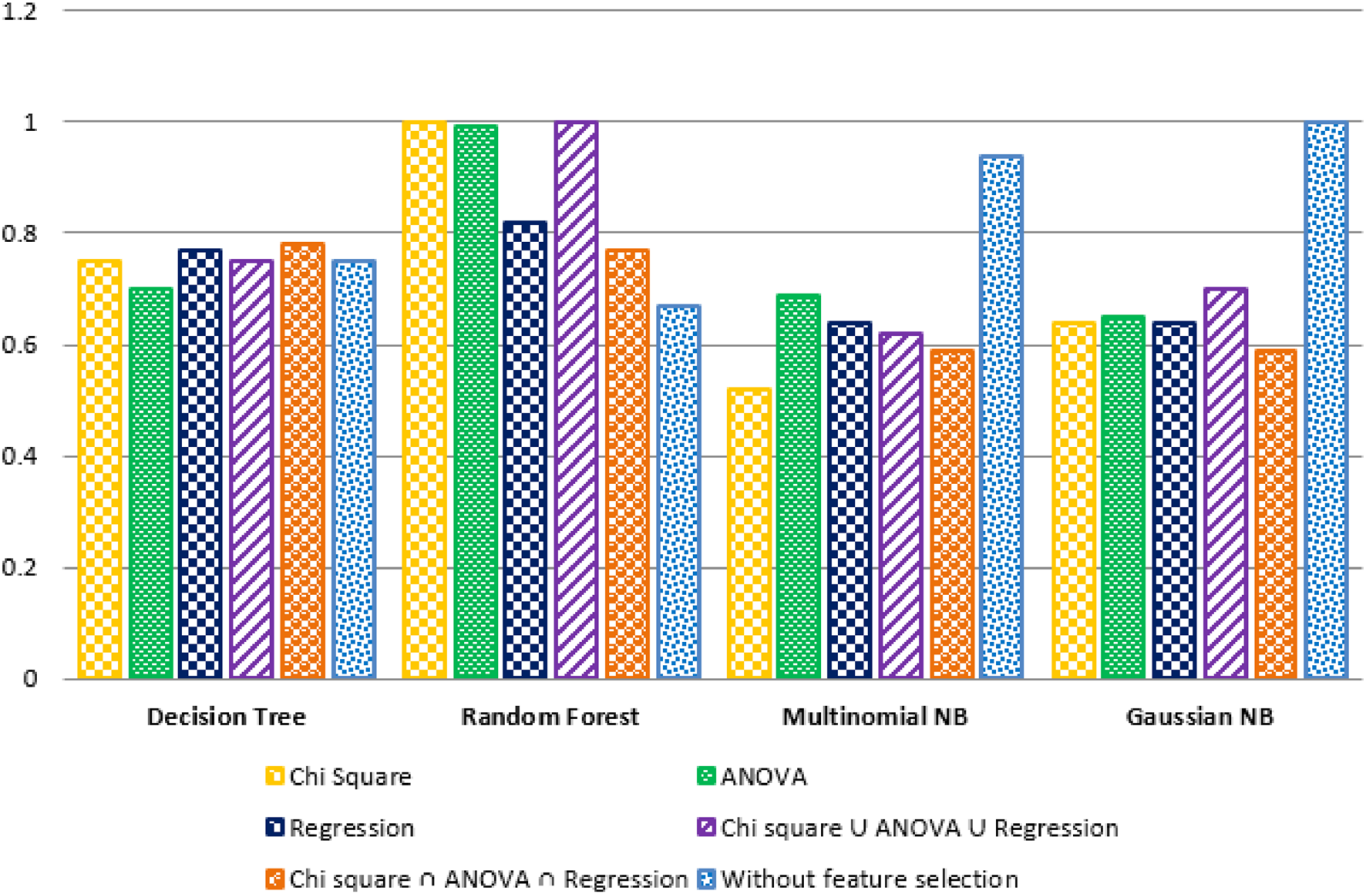
Comparative result of the different classifiers considering F1 score (feature selection and non-feature selection).

Secondly, we compared the prediction result of each classifier according to their weighted average F1 score (y-axis), as shown in Fig. 8. It depicts that, similar to the precision result, Random Forest classifier was found effective for {chi-square} and {chi-square ∪ Two-way ANOVA ∪ Regression} features with maximum weighted average precision. The Decision Tree had the best result for {chi-square ∩ Two-way ANOVA ∩ Regression} feature selection technique with a maximum of 0.77 F1 scores (1.0). Besides, the Decision Tree provided a satisfactory result for {chi-square ∩ Two-way ANOVA ∩ Regression} feature selection technique with a maximum weighted average F1 score (0.78). In contrast, Multinomial Naïve Bayes and Gaussian Naïve Bayes performed better without a feature selection process (0.94 and 1.0 Weighted Avg. F1 score, respectively).

### Evaluation-2

After evaluating the classifier's result through weighted average precision and F1 measure (evaluation-1), we compared the classifier's result through classification accuracy, sensitivity, and specificity. Table 3 represents the outcome of classification accuracy, sensitivity, and specificity of three classifiers: Decision Tree, Random Forest, and Naïve Bayes with feature selection and without a feature selection process. It represents that for chi-square, Two-way ANOVA, and {chi-square ∪ Two-way ANOVA ∪ Regression} selective features, Random Forest classifier provided the highest classification accuracy (1.0, 0.992, 1.0) with high sensitivity (1.0, 1.0, and 1.0) and high specificity (1.0, 1.0, 1.0). In contrast, for {chi-square ∩ Two-way ANOVA ∩ Regression} selective features, the Random Forest classifier did not perform well. Besides, none of the classifiers was effective for regression analysis features. Furthermore, the Decision Tree classifier performed well for {chi-square ∩ Two-way ANOVA ∩ Regression} selective features with maximum accuracy (0.80), sensitivity (0.95), and specificity (0.68). Multinomial Naïve Bayes and Gaussian Naïve Bayes perform well for the non-feature selection model. It achieves the maximum classification accuracy (0.93, 1.0), sensitivity (1.0), and specificity (1.0).

**Table 3:** Accuracy, Sensitivity, and Specificity of classifiers for several feature selection and non-feature selection processes.

Classifier	Chi-square features			Two-way ANOVA test features			Regression analysis features		
	Classification Accuracy	Sensitivity	Specificity	Classification Accuracy	Sensitivity	Specificity	Classification Accuracy	Sensitivity	Specificity
Decision tree	0.75	0.84	0.40	0.73	0.89	0.47	0.78	0.87	0.53
Random forest	1.0	1.0	1.0	0.992	1.0	1.0	0.83	0.95	0.73
Multinomial Naïve Bayes	0.47	0.96	0.60	0.73	0.92	0.46	0.72	0.97	0.60
Gaussian Naïve Bayes	0.70	0.96	0.40	0.72	1.0	0.0	0.78	0.97	0.60

	Chi-square ∪ two-way ANOVA ∪ regression			Chi-square ∩ Two-way ANOVA ∩ regression			Without feature selection		
Decision tree	0.75	0.81	0.48	0.80	0.95	0.68	0.77	0.92	0.55
Random forest	1.0	1.0	1.0	0.78	0.89	0.62	0.75	0.98	0.75
Multinomial Naïve Bayes	0.59	0.87	0.36	0.66	0.93	0.50	0.93	1.0	1.0
Gaussian Naïve Bayes	0.60	0.87	0.33	0.66	0.93	0.50	1.0	1.0	1.0

## Result and discussion

**Figure 9:**
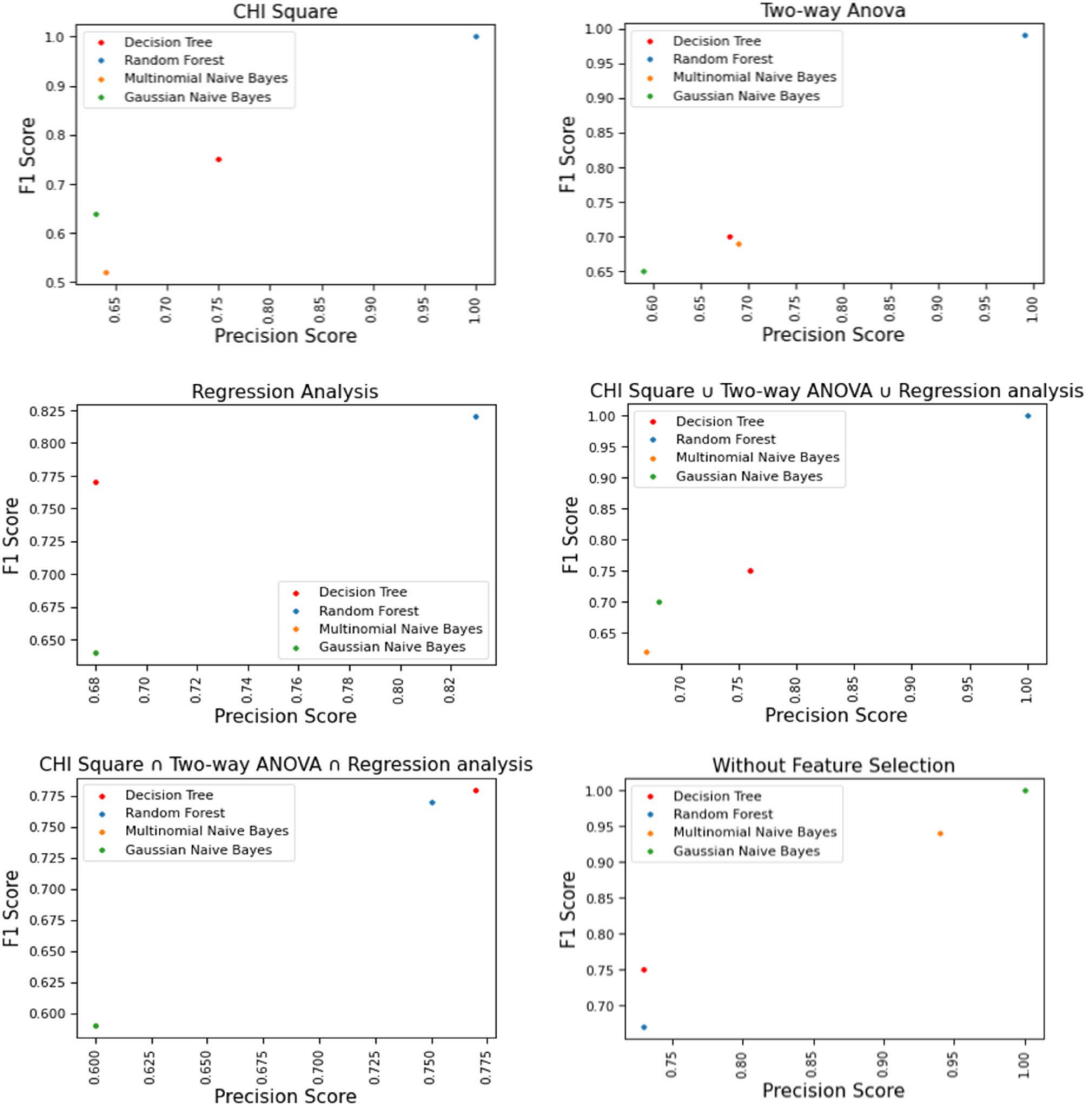
Performance of several machine learning algorithms and feature selection techniques.

Evaluation 1 concluded that precision measure and F1 score are equally important for evaluating any crash severity prediction model. Here, we considered both the precision measure and F1 score of each classifier, considering feature selection and non-feature selection techniques. Figure 9 shows that the Random Forest classifier performs better in feature selection than the other four classifiers. Besides, Decision Tree classifies high precision measure and F1 score for {chi-square ∩ Two-way ANOVA ∩ Regression} with the feature selection process. Furthermore, Gaussian Naïve Bayes performs better than other classifiers for the non-feature selection process. Thus, according to precision and F1 score, Random Forest was the best classifier of this crash severity prediction model.

**Figure 10:**
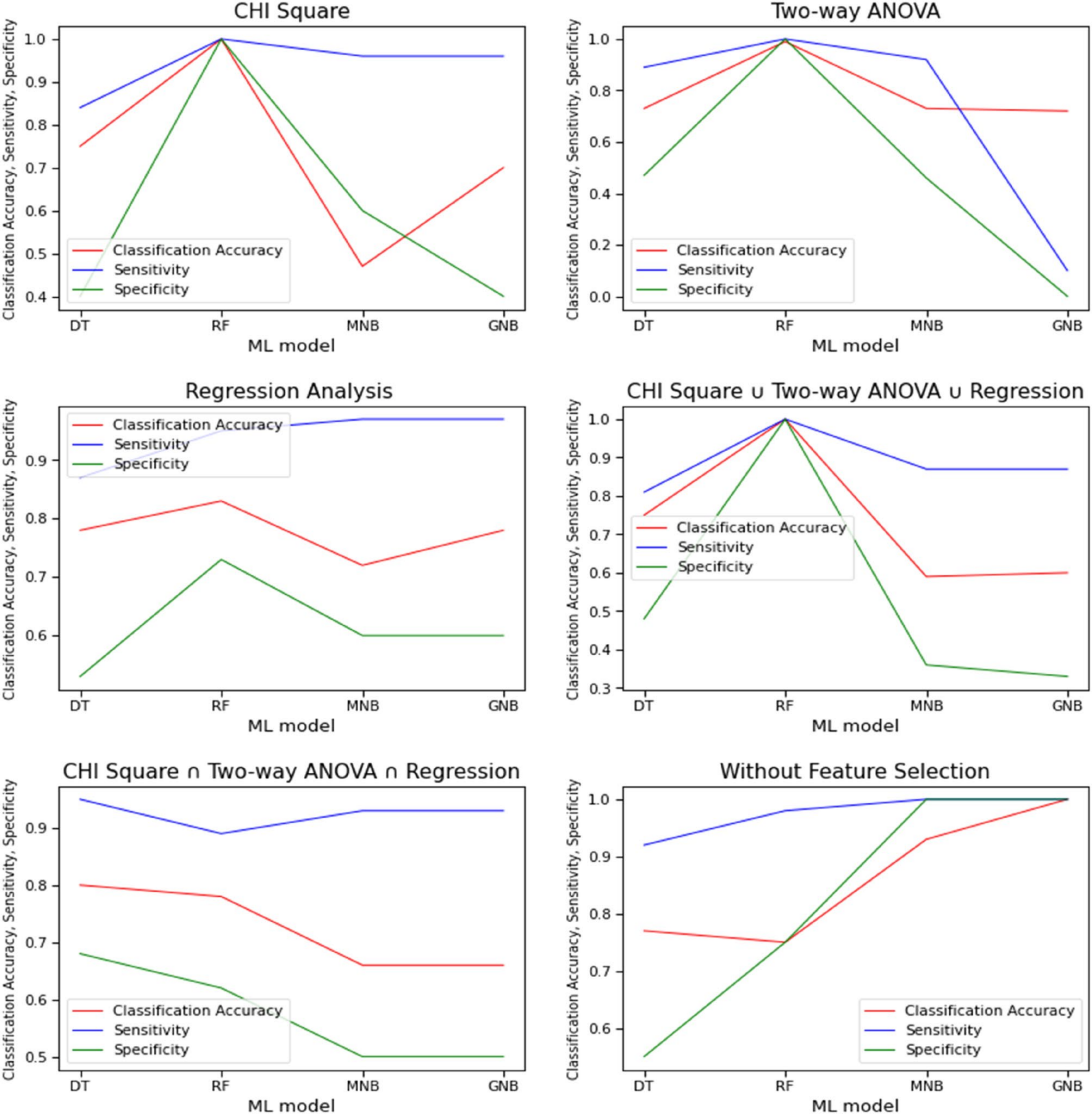
Performance of several machine learning algorithms and feature selection techniques considering accuracy, sensitivity, and specificity.

According to the second evaluation result, classification accuracy, sensitivity, and specificity were equally important for evaluating crash severity prediction models. Therefore, in this study, we emphasized high classification accuracy, sensitivity, and specificity together to evaluate the classifier's effectiveness. Figure 10 shows that the Random Forest classifier performed well for three feature selection processes: chi-square, ANOVA, and {chi-square ∪ Two-way ANOVA ∪ Regression} than other models. The Decision Tree classifier performed better for one feature selection process. In contrast, Multinomial Naïve Bayes and Gaussian Naïve Bayes were effective with maximum classification accuracy, sensitivity, and specificity for non-feature selection or without a feature selection process. Random Forest performed best for the majority of the feature selection process. Regarding chi-square and ANOVA, the result showed that Random Forest performance was comparatively better than other models. However, the analysis was not conclusive in identifying the best classifier in the current study context in terms of regression.

The importance of the feature selection process was described through the comparison result of classification accuracy, sensitivity, and specificity for both feature selection and non-feature selection. The comparison result depicts that the Random Forest classifier had superiority over the feature selection process. The Decision Tree had excellence for {chi-square ∩ Two-way ANOVA ∩ Regression} feature selection process over without feature selection process. In contrast, for the Naïve Bayes classifier, none of the feature selections performed well over the non-feature selection process. Therefore, it concludes that feature selection is vital for Random Forest and Decision Tree but not for the Naïve Bayes classifier.

## List of crash factors

Moreover, the evaluation result depicted that feature selection was effective and helped to improve the classification and prediction result^13,15,16,21,27^. Among several feature selection techniques, chi-square and Two-way ANOVA were found as effective in improving the accuracy of our crash severity and risk factor identification model. Therefore, features identified by these two techniques were considered the most responsible factors for influencing crashes. The identified nine risk factors are: Day of crash, Residential location, Vehicle type, License type, Seat belts, Gender, Time of crash, Road surface type, and Road classification. The Standard Deviation statistic was used here to evaluate the identified factors. The detailed results of SD are shown in Table A.1 of the Appendix section. The result specific to these features are discussed in the following sections:

### Day of crash

Day of crash was found to be one of the most significant factors of crash severity outcome. SD Result suggests that among 12 months, February (SD-1.68), June (SD-1.631), and October (SD-1.625) experienced a significant number of severe injury crashes, whereas February (SD-0.653) experienced more severe injury crashes than the rest of the months. In contrast, March (SD-0.137) experienced a significant number of non-fatal injury crashes than other months of the year. So, it concluded that severe injury crashes occur more frequently than extremely severe and non-fatal injury crashes throughout the year. To control the injury, traffic safety authorities should control the vehicle on the road more carefully in these months.

### Residential location

From the statistics of 47 places crash data, Dhaka is the place where the probability of severe crashes was higher (SD-5.916) than other places in the country. Also, Bagerhat (SD-1.50), Chattogram (SD-1.50), and Faridpur (SD-1.74) experienced a significant number of extremely severe crashes that would increase the probability of future extremely severe crashes. Gazipur likely had fewer severe and extremely severe crashes but experienced several non-fatal crashes than other places. Traffic authorities should increase their monitoring in this area to control road injuries.

### Vehicle type

Based on the findings, we can observe that Auto Rickshaw (SD-6.075), Footpath (SD-9.099), Motorcycle (SD-9.697), Mini-Truck (SD-2.993), and Truck (SD-4.191) were found responsible for increasing the probability of severe crashes and reducing the likelihood of non-fatal or extremely severe crashes. In contrast, maximum severe crashes occurred for Bus (SD-5.046) and Microbus (SD-2.554). In most cases, old buses and microbuses were associated with most crashes. Therefore, traffic authorities should focus on the fitness of vehicles to reduce accidents. Tractors, cars, and Bicycles identify with being involved in fewer medium and mild injury crashes.

### License type

License type plays a vital role in crash severity analysis. In this study, license type is categorized into P-professional (those who have licensed) and NP-nonprofessional (those who have no license). The statistic showed that severe crashes (SD-12.32) occur more frequently for nonprofessional drivers than professional drivers (SD-9.599). But for the professional driver, non-fatal and extremely severe crashes were higher than a nonprofessional driver with SD-0.533 for non-fatal and SD-4.799 for extremely severe crashes. These may be due to not abiding by the traffic rules, speed limits, or overconfident attitudes. Traffic authorities should take steps to increase awareness about traffic rules and their importance.

### Seat belts

One of the important factors for road crashes is seat belts. The analysis showed that not wearing seat belts was more likely to cause severe, non-fatal, and extremely severe crashes with SD 15.401, 0.393, 4.477 (Appendix Table A.1), respectively. Government and transportation safety policymakers should encourage people to wear seat belts to minimize unexpected crash consequences.

### Gender

Male drivers were more likely to be involved in severe and extremely severe crashes with SD-14.47 and 4.943, respectively.

### Time of crash

To identify the time of crashes, we considered daytime and nighttime to investigate our crash data. Statistics showed that for severe (SD-13.151) and extremely severe (SD-4.402) crashes, the probability of crash occurrence in the daytime was higher than at night. Generally, traffic volume was more in the daylight than at night time. Therefore, people are running out of time and intend to drive at high speed or lack concern about traffic rules that might increase crashes. Traffic authorities should improve their monitoring at night to control crashes on the road.

### Road surface type

We focused on five types of road surfaces such as Diamond Interchange, Zig Zag Road, J Turns, Circular Road, and Normal Road to analyze crash data. We found that severe and extremely severe crashes were more likely to occur on Normal Road surfaces compared to other road surfaces with SD11.03 and 3.497, respectively. Also, the Circular roadway surface was responsible for a large number of non-fatal crashes with SD 0.328.

### Road classification

We found that crashes were more likely to be severe when considering National Highway (NH) with 8.895 SD. Compared to other road classes: extremely severe crashes were more likely to happen in Regional Highway (RH) road class with SD 3.054. In contrast, Union Roads was more likely to be responsible for non-fatal crashes.

## Implications

Nowadays, crash severity prediction is a global issue for the government and road safety authorities. In this study, machine learning techniques with a feature selection process efficiently predict crash severity and help to identify responsible factors associated with crashes. Predicting crash severity levels may be considered crucial information for assessing crash risk factors. Using this model, the road safety authority can identify the responsible factors for non-fatal, severe, and extremely severe risk crashes, which is sometimes difficult to understand by road safety professionals and other policymakers. Therefore, this study might be helpful for accident prevention. However, it is noteworthy that the prediction result is not a complete absolute prediction and may vary in different situations. Therefore, safety professionals should carefully monitor the responsible factors identified in this study. Moreover, this study would assist safety professionals in understanding the unrevealed information and predicting associate attributes that influence crash severity.

## Conclusion and future work

In this paper, we present a machine learning-based crash severity prediction model from the perspective of road crashes in Bangladesh. This model analyzes four hundred and forty-one driver crashes in different parts of Bangladesh. The study went through data collecting, data preprocessing, crash data coding, three machine learning model implementations, comprehensive data analysis, and identifying contributing factors to crash severity. After implementing and analyzing, we have the following major findings:

1. Feature selection is prominent with machine learning models to identify the responsible factors and improve model performance. Chi-square and Two-way ANOVA have been found significant in examining the crash data.
2. Machine learning models are beneficial to uncover new insights into heterogeneous crash datasets. Random Forest and Decision Tree seem to be effective options for predicting crash severity for better prediction performance.
3. The features identified as responsible factors in our crash severity prediction model are the 'day of crash', *'*residential location', 'vehicle type', 'license type', 'seat belts', 'gender', 'time of crash', 'road surface type', and 'road classification'.
4. According to the research findings (crash factors), it could be argued that people might not have adequate knowledge of safe driving and perhaps are unwilling to follow traffic rules, such as refusing to wear seat belts, driving without having a license, using unfit vehicles, lack knowledge about traffic rules associated with road types (National Highway (NH) and Regional Highway (RH)), road surface types (Diamond Interchange, Zig Zag Road, J Turns, Circular Road, and Normal Road), area type (commercial and residential area), and speed limits during day time and night time. Police and government authorities should spread awareness about traffic rules and safety issues, impose traffic rules more strictly and monitor vehicle movement and vehicle fitness on a regular basis. It might help to reduce the upward trend of crashes in Bangladesh.

Future work would consider other machine learning and neural network models. For instance, we want to work with different types of machine learning algorithms: Support Vector Machine^12^, Logistic Regression^21^, K-Nearest Neighbor^18^, and neural networks models: Artificial Neural Network^15^, Feedforward Neural Network^6^, and Multilayer Perceptron^10^. We intend to increase the dimension of the dataset, considering other factors that are likely to be responsible for crashes. Therefore, we will collect data from different sources (accident reports and police feedback) and focus on data imbalance handling^18^, which might improve the accuracy of our future studies. Additionally, the compiled data will be validated against other official open-source data, which is one of the prime concerns of future work.

## Data availability

All data generated or analyzed during this study are included in this published article. It is also available in—BD_Road_Crash_Data.

## Supplementary Information

Supplementary Information 1.

Supplementary Information 2.
