# Supplementary material for: Crash severity analysis and risk factors identification based on an alternate data source: a case study of developing country
Source: Sci Rep. 2022 Dec 8;12:21243. doi: 10.1038/s41598-022-25361-5 (PMC9732348; doi:10.1038/s41598-022-25361-5)
Supplement: Supplementary file 1 — Supplementary Information 1. [file 41598_2022_25361_MOESM1_ESM.docx]

# APPENDIX

**Table A.1: Descriptive Statistics of responsible feature with crash ratio and injury severity level**

| **Crash factors with variables details** | **Crash ratio** | | | **Injury severity** | | | | | | |
| --- | --- | --- | --- | --- | --- | --- | --- | --- | --- | --- |
|  |  |  |  | **Severe** | | **Non-fatal** | | | **Extremely Severe** | |
|  | **Observed** | **mean** | **SD** | **mean** | **SD** | **mean** | | **SD** | **mean** | **SD** |
| **Vehicle type** | | | | | | | | | | |
| Auto Rickshaw | 78 | 0.176 | 3.710 | 0.692 | 6.075 | 0.012 | | 0.112 | 0.294 | 2.587 |
| BiCycle | 1 | 0.002 | 0.047 | 1 | 0 | -- | | -- | -- | -- |
| Bus | 75 | 0.170 | 3.567 | 0.360 | 3.096 | 0.054 | | 0.458 | 0.586 | 5.046 |
| Car | 1 | 0.002 | 0.047 | -- | -- | -- | | -- | 1 | 0 |
| Footpath | 99 | 0.224 | 4.708 | 0.919 | 9.099 | 0.030 | | 0.300 | 0.050 | 0.500 |
| MicroBus | 29 | 0.065 | 1.379 | 0.448 | 2.372 | 0.068 | | 0.365 | 0.482 | 2.554 |
| Mini-Truck | 15 | 0.034 | 0.713 | 0.80 | 2.993 | -- | | -- | 0.20 | 0.748 |
| Motorcycle | 114 | 0.258 | 5.422 | 0.912 | 9.697 | -- | | -- | 0.087 | 0.932 |
| Tractor | 1 | 0.002 | 0.047 | 1 | 0 | -- | | -- | -- | -- |
| Truck | 28 | 0.063 | 1.331 | 0.821 | 4.191 | -- | | -- | 0.178 | 0.911 |
| **Time of crash** | | | | | | | | | | |
| Day | 321 | 0.727 | 15.268 | 0.735 | 13.151 | 0.018 | | 0.334 | 0.246 | 4.402 |
| Night | 120 | 0.272 | 5.707 | 0.750 | 8.181 | 0.033 | | 0.363 | 0.216 | 2.363 |
| **Gender** | | | | | | | | | | |
| Male | 392 | 0.889 | 18.645 | 0.732 | 14.47 | 0.017 | 0.353 | | 0.250 | 4.943 |
| Female | 49 | 0.112 | 2.330 | 0.795 | 5.514 | 0.061 | 0.424 | | 0.142 | 0.989 |
| **Road classification** | | | | | | | | | | |
| RH | 141 | 0.319 | 6.706 | 0.751 | 8.895 | 0.007 | 0.083 | | 0.241 | 2.853 |
| NH | 102 | 0.231 | 4.851 | 0.676 | 6.798 | 0.019 | 0.197 | | 0.303 | 3.054 |
| UpR | 61 | 0.138 | 2.901 | 0.770 | 5.968 | 0.032 | 0.254 | | 0.196 | 1.523 |
| ZR | 52 | 0.117 | 2.473 | 0.750 | 5.356 | 0.019 | 0.137 | | 0.230 | 1.648 |
| UnR | 46 | 0.104 | 2.188 | 0.717 | 4.812 | 0.086 | 0.583 | | 0.195 | 1.312 |
| VR | 39 | 0.088 | 1.855 | 0.820 | 5.058 | -- | -- | | 0.179 | 1.106 |
| **Number of lanes** | | | | | | | | | | |
| Lane-1 | 146 | 0.331 | 6.944 | 0.767 | 9.237 | 0.041 | 0.494 | | 0.191 | 2.309 |
| Lane-2 | 52 | 0.117 | 2.470 | 0.750 | 5.356 | 0.019 | 0.137 | | 0.230 | 1.648 |
| Lane-4 | 141 | 0.319 | 6.706 | 0.751 | 8.895 | 0.007 | 0.083 | | 0.241 | 2.853 |
| Lane-6 | 102 | 0.231 | 4.851 | 0.676 | 6.798 | 0.019 | 0.197 | | 0.303 | 3.054 |
| **Road Surface type** | | | | | | | | | | |
| Circular Road | 36 | 0.081 | 1.712 | 0.694 | 4.108 | 0.055 | 0.328 | | 0.250 | 1.479 |
| Diamond Interchange | 36 | 0.081 | 1.712 | 0.750 | 4.437 | -- | -- | | 0.250 | 1.479 |
| J Turns | 50 | 0.113 | 2.378 | 0.70 | 4.90 | 0.02 | 0.14 | | 0.28 | 1.96 |
| Normal | 220 | 0.499 | 10.46 | 0.745 | 11.03 | 0.018 | 0.269 | | 0.236 | 3.497 |
| Zig Zag Road | 99 | 0.224 | 4.708 | 0.757 | 7.499 | 0.030 | 0.300 | | 0.212 | 2.099 |
| **Weather** | | | | | | | | | | |
| Gloomy | 142 | 0.321 | 6.754 | 0.739 | 8.780 | 0.042 | 0.501 | | 0.219 | 2.592 |
| Hot | 141 | 0.319 | 6.706 | 0.730 | 8.643 | 0.014 | 0.167 | | 0.255 | 3.021 |
| Rainy | 96 | 0.217 | 4.566 | 0.739 | 7.208 | 0.020 | 0.203 | | 0.239 | 2.335 |
| Sunny | 62 | 0.140 | 2.949 | 0.758 | 5.920 | -- | -- | | 0.241 | 1.889 |
| **Lighting** | | | | | | | | | | |
| Light | 313 | 0.709 | 14.88 | 0.738 | 13.03 | 0.019 | 0.338 | | 0.242 | 4.288 |
| Grey | 76 | 0.172 | 3.614 | 0.723 | 6.267 | 0.039 | 0.341 | | 0.236 | 2.051 |
| Dark | 52 | 0.117 | 2.473 | 0.769 | 5.493 | 0.019 | 0.137 | | 0.211 | 1.510 |
| **Driver Age** | | | | | | | | | | |
| Middle-aged Adult | 200 | 0.453 | 9.513 | 0.720 | 10.156 | 0.035 | 0.493 | | 0.245 | 3.456 |
| Young Adult | 132 | 0.299 | 6.278 | 0.757 | 8.670 | 0.015 | 0.173 | | 0.227 | 2.601 |
| Older Adult | 109 | 0.247 | 5.184 | 0.752 | 7.818 | 0.009 | 0.095 | | 0.238 | 2.478 |
| **License type** | | | | | | | | | | |
| NP | 217 | 0.492 | 10.321 | 0.838 | 12.326 | 0.009 | 0.135 | | 0.152 | 2.235 |
| P | 224 | 0.507 | 10.654 | 0.642 | 9.599 | 0.035 | 0.533 | | 0.321 | 4.799 |
| **Residential location** | | | | | | | | | | |
| Ashulia | 3 | 0.006 | 0.142 | 0.67 | 0.940 | -- | -- | | 0.33 | 0.473 |
| Bagerhat | 10 | 0.022 | 0.475 | 0.50 | 1.50 | -- | -- | | 0.50 | 1.50 |
| Bandarban | 2 | 0.004 | 0.095 | 0.50 | 0.50 | -- | -- | | 0.50 | 0.50 |
| Barisal | 5 | 0.011 | 0.237 | 0.60 | 1.20 | 0.20 | 0.40 | | 0.20 | 0.40 |
| Bhola | 5 | 0.011 | 0.237 | 0.80 | 1.60 | -- | -- | | 0.20 | 0.40 |
| Bogra | 17 | 0.038 | 0.808 | 0.764 | 3.05 | 0.05 | 0.237 | | 0.176 | 0.706 |
| Brahmanbaria | 10 | 0.022 | 0.475 | 0.70 | 2.10 | 0.10 | 0.30 | | 0.20 | 0.60 |
| Chadpur | 3 | 0.006 | 0.142 | 0.333 | 0.473 | -- | -- | | 0.67 | 0.940 |
| Chapainnawabganj | 1 | 0.002 | 0.047 | -- | -- | -- | -- | | 1.0 | 0.00 |
| Chattogram | 10 | 0.022 | 0.475 | 0.50 | 1.50 | -- | -- | | 0.50 | 1.50 |
| Chuadanga | 1 | 0.002 | 0.047 | 1.0 | 0.0 | -- | -- | | -- | -- |
| Comilla | 12 | 0.027 | 0.570 | 0.583 | 1.934 | -- | -- | | 0.417 | 1.381 |
| Cox's Bazar | 3 | 0.006 | 0.142 | 1.0 | 1.414 | -- | -- | | -- | -- |
| Dhaka | 47 | 0.106 | 2.235 | 0.872 | 5.916 | 0.021 | 0.144 | | 0.106 | 0.721 |
| Dinajpur | 10 | 0.022 | 0.475 | 0.70 | 2.10 | -- | -- | | 0.30 | 0.90 |
| Faridpur | 7 | 0.015 | 0.332 | 0.285 | 0.700 | -- | -- | | 0.714 | 1.749 |
| Feni | 5 | 0.011 | 0.237 | 0.80 | 1.60 | -- | -- | | 0.20 | 0.40 |
| Fouzdarhat | 1 | 0.002 | 0.047 | 1.0 | 0.0 | -- | -- | | -- | -- |
| Gaibandha | 4 | 0.009 | 0.190 | 1.0 | 1.732 | -- | -- | | -- | -- |
| Gazipur | 14 | 0.031 | 0.665 | 0.714 | 2.575 | 0.285 | 1.030 | | -- | -- |
| Gobindaganj | 1 | 0.002 | 0.047 | 1.0 | 0.0 | -- | -- | | -- | -- |
| Godagari | 1 | 0.002 | 0.047 | 1.0 | 0.0 | -- | -- | | -- | -- |
| Gopalganj | 16 | 0.036 | 0.761 | 0.812 | 3.146 | -- | -- | | 0.187 | 0.726 |
| Hathazari | 1 | 0.002 | 0.047 | -- | -- | 1.0 | 0.0 | | -- | -- |
| Hili | 1 | 0.002 | 0.047 | 1.0 | 0.0 | -- | -- | | -- | -- |
| Hobiganj | 9 | 0.020 | 0.428 | 1.0 | 2.828 | -- | -- | | -- | -- |
| Jamalpur | 6 | 0.013 | 0.285 | 1.0 | 2.236 | -- | -- | | -- | -- |
| Jatrabari | 1 | 0.002 | 0.047 | 1.0 | 0.0 | -- | -- | | -- | -- |
| Jaypurhat | 2 | 0.004 | 0.095 | -- | -- | -- | -- | | 1.0 | 1.0 |
| Jessore | 15 | 0.034 | 0.713 | 0.733 | 2.743 | 0.133 | 0.498 | | 0.133 | 0.498 |
| Jhenaidah | 10 | 0.022 | 0.475 | 0.90 | 2.70 | 0.10 | 0.30 | | -- | -- |
| Keraniganj | 2 | 0.004 | 0.095 | 1.0 | 1.0 | -- | -- | | -- | -- |
| Khagrachhari | 1 | 0.002 | 0.047 | 1.0 | 0.0 | -- | -- | | -- | -- |
| Khulna | 5 | 0.011 | 0.237 | 0.60 | 1.20 | 0.40 | 0.80 | | -- | -- |
| Kurigram | 4 | 0.009 | 0.190 | 0.75 | 1.299 | -- | -- | | 0.25 | 0.433 |
| Kishoreganj | 3 | 0.006 | 0.142 | 0.67 | 0.940 | -- | -- | | 0.33 | 0.473 |
| Kushtia | 5 | 0.011 | 0.237 | 0.80 | 1.60 | -- | -- | | 0.20 | 0.40 |
| Lakshmipur | 6 | 0.013 | 0.285 | 0.67 | 1.489 | -- | -- | | 0.33 | 0.746 |
| Lalmonirhat | 3 | 0.006 | 0.142 | 1.0 | 1.414 | -- | -- | | -- | -- |
| Madaripur | 5 | 0.011 | 0.237 | 0.60 | 1.20 | -- | -- | | 0.40 | 0.80 |
| Magura | 7 | 0.015 | 0.332 | 1.0 | 2.449 | -- | -- | | -- | -- |
| Manikganj | 2 | 0.004 | 0.095 | 1.0 | 1.0 | -- | -- | | -- | -- |
| MoulviBazar | 4 | 0.009 | 0.190 | 1.0 | 1.932 | -- | -- | | -- | -- |
| Munshiganj | 7 | 0.015 | 0.332 | 0.571 | 1.399 | -- | -- | | 0.428 | 1.050 |
| Mymensingh | 1 | 0.002 | 0.047 | 1.0 | 0.0 | -- | -- | | -- | -- |
| Naogaon | 9 | 0.020 | 0.428 | 0.78 | 2.199 | -- | -- | | 0.222 | 0.628 |
| Narayanganj | 13 | 0.029 | 0.618 | 0.769 | 2.664 | -- | -- | | 0.230 | 0.799 |
| Narsingdi | 5 | 0.011 | 0.237 | 0.80 | 1.60 | -- | -- | | 0.20 | 0.40 |
| Natore | 8 | 0.018 | 0.380 | 0.625 | 1.653 | -- | -- | | 0.375 | 0.992 |
| Netrokona | 2 | 0.004 | 0.095 | 0.5 | 0.5 | -- | -- | | 0.5 | 0.5 |
| Nilphamari | 2 | 0.004 | 0.095 | 1.0 | 1.0 | -- | -- | | -- | -- |
| Noakhali | 9 | 0.020 | 0.428 | 0.33 | 0.943 | 0.111 | 0.314 | | 0.555 | 1.571 |
| Pabna | 5 | 0.011 | 0.237 | 0.80 | 1.60 | -- | -- | | 0.20 | 0.40 |
| Panchagar | 3 | 0.006 | 0.142 | 0.67 | 0.940 | -- | -- | | 0.333 | 0.471 |
| Patuakhali | 3 | 0.006 | 0.142 | 1.0 | 1.414 | -- | -- | | -- | -- |
| Pirojpur | 3 | 0.006 | 0.142 | 1.0 | 1.414 | -- | -- | | -- | -- |
| Rahobol | 1 | 0.002 | 0.047 | 1.0 | 0.0 | -- | -- | | -- | -- |
| Rajbari | 6 | 0.013 | 0.285 | 0.67 | 1.489 | -- | -- | | 0.333 | 0.745 |
| Rajshahi | 6 | 0.013 | 0.285 | 0.67 | 1.489 | -- | -- | | 0.333 | 0.745 |
| Rampal | 1 | 0.002 | 0.047 | 1.0 | 0.0 | -- | -- | | -- | -- |
| Rangamati | 4 | 0.009 | 0.190 | 1.0 | 1.732 | -- | -- | | -- | -- |
| Rangpur | 5 | 0.011 | 0.237 | 0.80 | 1.60 | -- | -- | | 0.20 | 0.40 |
| Satkhira | 5 | 0.011 | 0.237 | 1.0 | 2.0 | -- | -- | | -- | -- |
| Savar | 2 | 0.004 | 0.095 | 1.0 | 1.0 | -- | -- | | -- | -- |
| Shariatpur | 1 | 0.002 | 0.047 | -- | -- | -- | -- | | 1.0 | 0.0 |
| Shatkhira | 4 | 0.004 | 0.190 | 0.75 | 1.299 | -- | -- | | 0.25 | 0.433 |
| Sherpur | 4 | 0.004 | 0.190 | 0.75 | 1.299 | -- | -- | | 0.25 | 0.433 |
| Siddhirganj | 1 | 0.002 | 0.047 | 1.0 | 0.0 | -- | -- | | -- | -- |
| Sirajganj | 18 | 0.040 | 0.856 | 0.722 | 2.977 | -- | -- | | 0.278 | 1.145 |
| Sreenagar | 1 | 0.002 | 0.047 | -- | -- | 1.0 | 0.0 | | -- | -- |
| Sunamganj | 3 | 0.006 | 0.142 | 0.333 | 0.473 | -- | -- | | 0.67 | 0.940 |
| Sylhet | 1 | 0.002 | 0.047 | 1.0 | 0.0 | -- | -- | | -- | -- |
| Tangail | 18 | 0.040 | 0.856 | 0.833 | 3.436 | 0.055 | 0.229 | | 0.111 | 0.458 |
| Thakurgaon | 9 | 0.020 | 0.428 | 0.67 | 1.884 | -- | -- | | 0.33 | 0.943 |
| Wazirpur | 1 | 0.002 | 0.047 | 1.0 | 0.0 | -- | -- | | -- | -- |
| **Seat Belts** | | | | | | | | | | |
| No | 412 | 0.934 | 19.59 | 0.759 | 15.401 | 0.019 | 0.393 | | 0.220 | 4.477 |
| Yes | 29 | 0.065 | 1.379 | 0.448 | 2.372 | 0.068 | 0.365 | | 0.482 | 2.554 |
| **Vehicle age** | | | | | | | | | | |
| 10 | 125 | 0.283 | 5.945 | 0.736 | 8.195 | 0.032 | 0.356 | | 0.232 | 2.583 |
| 20 | 117 | 0.265 | 5.565 | 0.760 | 8.192 | 0.017 | 0.184 | | 0.222 | 2.393 |
| 30 | 199 | 0.451 | 9.465 | 0.728 | 10.252 | 0.020 | 0.282 | | 0.251 | 3.535 |
